# *Neochloris* sp. PA3, an Oligotrophic Desert Oasis Microalga, Exhibits Differential Cytotoxicity and Apoptosis-Associated Effects in Human Cancer Cells

**DOI:** 10.3390/ijms27209258

**Published:** 2026-10-18

**Authors:** Maripaz Castillo-Roque, José Alberto Ramos-Silva, José Antonio Fuentes-Garibay, Martha Reyes-Becerril, Susana De la Torre-Zavala, Alan G. Hernández-Melgar, Mario Figueroa, Even Ramos-Hinojosa, Luis Jesús Galán-Wong, Hamlet Avilés-Arnaut

**Affiliations:** 1 Universidad Autónoma de Nuevo León, Facultad de Ciencias Biológicas, Instituto de Biotecnología, Avenida Universidad S/N, Ciudad Universitaria, San Nicolás de los Garza 66455, Mexico; maripazmcr_09@hotmail.com (M.C.-R.); antonio.fuentesgr@uanl.edu.mx (J.A.F.-G.); susana.delatorrezv@uanl.edu.mx (S.D.l.T.-Z.); evenramosh@gmail.com (E.R.-H.); ljgw55@gmail.com (L.J.G.-W.); 2 Immunology & Vaccinology Group: Phytochemistry Applied to Animal Health, Centro de Investigaciones Biologicas del Noroeste, S.C., Av. Instituto Politecnico Nacional 195, Playa Palo de Santa Rita Sur, La Paz 23096, Mexico; mreyes04@cibnor.mx; 3 Facultad de Química, Universidad Nacional Autónoma de México, Ciudad de México 04510, Mexico; alan_hernandez@quimica.unam.mx (A.G.H.-M.); mafiguer@unam.mx (M.F.)

**Keywords:** microalgae, antioxidant properties, differential cytotoxicity, apoptosis-associated cell death, in vitro anticancer activity

## Abstract

Microalgae are increasingly recognized as valuable sources of natural products with potential applications in cancer prevention and therapy due to their ability to produce structurally diverse bioactive metabolites. In the present study, methanolic extracts from microalgal strains isolated from the oligotrophic desert oasis of the Cuatro Ciénegas Basin (Mexico) were evaluated for their antioxidant activity and in vitro cytotoxic effects against human breast (MCF7), colon (Caco2), prostate (DU145), leukemia (MOLT-4), and ovarian (OVCAR-3) cancer cell lines. The isolates were identified by *rbcL* gene sequencing and phylogenetic analysis as members of the genera *Asterarcys*, *Chlorella*, and *Neochloris*. Antioxidant properties were evaluated using complementary in vitro assays, whereas cytotoxicity was assessed using the MTT assay and apoptosis-associated cellular responses were investigated by morphological analysis, acridine orange/ethidium bromide staining, and measurement of executioner caspase-3/7 activity. *Neochloris* sp. PA3 showed the highest total antioxidant capacity and detectable ferrous ion-chelating activity, whereas *Chlorella* sp. PS exhibited the strongest superoxide radical scavenging activity. Among the evaluated strains, the methanolic extract of *Neochloris* sp. PA3 exhibited the most favorable overall antiproliferative profile, with IC_50_ values of 17.1, 19.0, and 19.1 µg/mL against MCF7, Caco2, and DU145 cells, respectively, while displaying markedly lower cytotoxicity toward the non-tumorigenic Vero and Detroit 548 cell lines, indicating differential cytotoxicity between the tested cancer and non-tumorigenic cell models. The extract induced time-dependent reductions in cell viability, morphological and nuclear alterations consistent with apoptotic cell death, and increased executioner caspase-3/7 activity. Collectively, these findings provide preliminary in vitro evidence of differential cytotoxicity and an apoptosis-associated cellular response in *Neochloris* sp. PA3-treated cancer cells.

## 1. Introduction

Cancer remains one of the leading causes of morbidity and mortality worldwide and continues to represent a major public health challenge. According to the most recent estimates from the Global Cancer Observatory (GLOBOCAN), approximately 20 million new cancer cases and 9.7 million cancer-related deaths occurred worldwide in 2022, with breast, colorectal, and prostate cancers among the most frequently diagnosed malignancies [1]. Although substantial advances have been achieved in surgery, radiotherapy, immunotherapy, and targeted therapies, chemotherapy continues to play a central role in the treatment of many solid tumors. However, the lack of selectivity of many chemotherapeutic agents frequently results in severe systemic toxicity, drug resistance, and limited therapeutic efficacy, highlighting the need for safer and more effective anticancer agents capable of selectively targeting malignant cells while minimizing damage to healthy tissues [2,3].

Natural products have historically constituted one of the most successful sources of therapeutic agents and continue to inspire the development of modern pharmaceuticals. Approximately two-thirds of all small-molecule anticancer drugs approved over the last four decades are either natural products, semisynthetic derivatives, or synthetic compounds inspired by natural molecular scaffolds [4]. Their remarkable structural diversity has enabled the discovery of compounds capable of modulating multiple cellular pathways involved in carcinogenesis, including oxidative stress, chronic inflammation, cell-cycle progression, angiogenesis, metastasis, and programmed cell death. Consequently, natural products have regained considerable interest as complementary or alternative therapeutic strategies in oncology, particularly those capable of simultaneously exerting antioxidant, anti-inflammatory, and anticancer activities [5]. Understanding the cellular mechanisms underlying these biological effects is essential for identifying promising candidates for future therapeutic development.

Among the various natural sources investigated for bioactive metabolites, microalgae have recently attracted considerable attention because of their extraordinary metabolic diversity and their capacity to synthesize compounds with significant pharmacological potential. These photosynthetic microorganisms produce a broad range of metabolites, including carotenoids, chlorophyll derivatives, polyunsaturated fatty acids, glycolipids, polysaccharides, phenolic compounds, sterols, peptides, and other secondary metabolites that have demonstrated antioxidant, anti-inflammatory, antimicrobial, and anticancer properties in experimental models [6,7,8]. Several studies have shown that microalgal extracts can inhibit cancer cell proliferation through multiple mechanisms, including induction of apoptosis, cell-cycle arrest, mitochondrial dysfunction, modulation of reactive oxygen species, and regulation of signaling pathways associated with tumor progression [9,10]. Owing to this remarkable chemical and biological diversity, microalgae are increasingly recognized as an underexplored reservoir of natural products with considerable potential for the discovery of novel anticancer agents.

Extreme environments have emerged as attractive targets for the discovery of novel natural products because microorganisms inhabiting these ecosystems are exposed to intense selective pressures that favor the evolution of unique metabolic adaptations. Oligotrophic habitats, characterized by chronic nutrient limitation, particularly phosphorus scarcity, impose environmental pressures that can influence microbial metabolism and may contribute to the production of specialized metabolites associated with stress tolerance, competition, and survival [11]. Consequently, microorganisms isolated from these environments have attracted increasing attention as promising sources of structurally diverse bioactive molecules with potential pharmaceutical applications. Despite this growing interest, freshwater microalgae from oligotrophic ecosystems remain considerably less explored than marine species, particularly regarding their cancer-related biological activity and underlying cellular mechanisms.

The Cuatro Ciénegas Basin (CCB), located within the Chihuahuan Desert in northern Mexico, is recognized as one of the world’s most exceptional freshwater oasis ecosystems. This protected basin comprises hundreds of springs and ponds characterized by extreme oligotrophy, unusual nitrogen-to-phosphorus stoichiometry, and an extraordinary diversity of endemic microorganisms [12,13]. These unique ecological conditions have persisted for millions of years, making the CCB an outstanding natural laboratory for studying microbial evolution and adaptation [14]. Previous investigations have focused primarily on the ecological and evolutionary significance of this ecosystem, whereas its microalgal diversity remains largely unexplored from a pharmacological perspective. Considering that environmental stress is frequently associated with the production of specialized metabolites, microalgae inhabiting this ecosystem may represent an untapped source of natural products with relevant biological activities.

Although the in vitro anticancer activities of several marine microalgae have been extensively investigated, relatively little is known about the biological activity of freshwater microalgae isolated from oligotrophic desert environments. Moreover, only a limited number of studies have examined differential cytotoxic responses between cancer and non-tumorigenic cell models and the cellular mechanisms underlying these biological effects. Therefore, the present study aimed to evaluate the antioxidant properties and in vitro cytotoxic activity of methanolic extracts obtained from microalgae isolated from different freshwater systems within the Cuatro Ciénegas Basin. The isolates were identified by *rbcL* gene sequencing and phylogenetic analysis, and their antioxidant properties were characterized using complementary in vitro assays assessing total antioxidant capacity, superoxide radical scavenging activity, and ferrous ion-chelating activity. Their cytotoxic activity was evaluated against human breast, colon, prostate, ovarian, and leukemia cancer cell lines, together with non-tumorigenic cell models to assess differential citotoxicity. The extract exhibiting the most favorable cytotoxic profile was further investigated through time-dependent viability, morphological analysis, acridine orange/ethidium bromide staining, and caspase-3/7 activity to characterize the cellular response associated with its cytotoxic effects.

## 2. Results

### 2.1. Morphological Characterization and Molecular Identification of Microalgal Strains

Four freshwater microalgal strains (MOJF-1, PA2, PA3, and PS) were morphologically and molecularly characterized. Bright-field microscopy revealed that all isolates belonged to the phylum Chlorophyta, although each strain exhibited distinctive morphological features that allowed their preliminary differentiation (Figure 1).

The MOJF-1 isolate consisted predominantly of spherical to slightly ovoid cells with relatively uniform dimensions and a well-defined cell wall. Most cells contained abundant green intracellular material occupying a large proportion of the cellular volume, with a centrally located translucent region suggestive of a pyrenoid. The PA2 isolate displayed a morphology similar to that of MOJF-1 but exhibited greater variability in cell shape, ranging from spherical to ellipsoidal cells, together with slightly smaller average cell size and a more elongated appearance.

In contrast, the PA3 isolate was characterized by markedly larger spherical cells with thick, well-defined cell walls and dense green intracellular contents that occupied nearly the entire cell. The PS isolate showed the smallest cells among the four strains, displaying predominantly spherical morphology, thin cell walls, and a comparatively lower intracellular volume occupied by chloroplast material. Unlike the other isolates, PS cells appeared more dispersed throughout the microscopic field and exhibited less morphological variability.

Partial *rbcL* sequences of 465, 528, 465, and 465 bp were obtained for isolates MOJF-1, PA2, PA3, and PS, respectively. BLASTn (ver 2.17.0) analysis showed that MOJF-1 had the highest sequence similarity to members of the genus *Asterarcys* (97.85% identity; 100% query coverage), whereas PA3 and PS showed the highest similarity to members of *Neochloris* (95.91% identity; 100% query coverage) and *Chlorella* (98.92% identity; 100% query coverage), respectively. For PA2, the highest-scoring BLASTn hit corresponded to *Asterarcys* sp. SC01 (93.49% identity; 64% query coverage), and several of the subsequent highest-scoring matches also belonged to the genus *Asterarcys*, although with relatively limited query coverage (59–71%). Phylogenetic analysis using representative sequences retrieved from GenBank (Tables S1–S3) further supported these genus-level assignments [15,16,17,18,19,20,21,22,23,24,25,26,27,28,29,30,31,32,33,34,35,36,37,38,39,40,41,42,43,44,45,46]. Maximum Likelihood phylogenetic reconstruction consistently grouped isolates MOJF-1 and PA2 within the genus *Asterarcys* (Scenedesmaceae), isolate PA3 within the genus *Neochloris* (Neochloridaceae), and isolate PS within the genus *Chlorella* (Chlorellaceae) (Figure 2 and Figures S1–S3). Because the analyses were based on partial *rbcL* sequences, which may provide insufficient resolution for unambiguous species-level discrimination among closely related chlorophytes, the isolates were conservatively identified at the genus level as *Asterarcys* sp. MOJF-1, *Asterarcys* sp. PA2, *Neochloris* sp. PA3, and *Chlorella* sp. PS.

The combined morphological and molecular analyses identified the isolates as *Asterarcys* sp. MOJF-1, *Asterarcys* sp. PA2, *Neochloris* sp. PA3, and *Chlorella* sp. PS. These taxonomically characterized strains were subsequently evaluated for their antiproliferative activity against human cancer cell lines.

### 2.2. Antioxidant Properties of the Microalgal Extracts

The antioxidant properties of the methanolic extracts obtained from the four microalgae were evaluated using three complementary assays to characterize different mechanisms of antioxidant activity, including total antioxidant capacity (TAC), superoxide radical scavenging activity, and ferrous ion-chelating activity (Figure 3).

The phosphomolybdenum assay revealed significant differences in total antioxidant capacity among the evaluated extracts (Figure 3A). *Neochloris* sp. PA3 exhibited the highest antioxidant capacity, reaching approximately 9.3 µmol ascorbic acid equivalents (AAE) g^−1^ dry weight (DW), followed by *Asterarcys* sp. PA2 (approximately 8.2 µmol AAE g^−1^ DW), *Chlorella* sp. PS (approximately 6.9 µmol AAE g^−1^ DW), and *Asterarcys* sp. MOJF-1 (approximately 5.2 µmol AAE g^−1^ DW). Significant differences were observed among all four extracts (*p* < 0.05), with PA3 exhibiting the greatest overall antioxidant capacity.

Superoxide radical scavenging activity also differed among the evaluated microalgae (Figure 3B). *Chlorella* sp. PS displayed the highest activity, scavenging approximately 82% of superoxide radicals, and was significantly more active than the other three extracts (*p* < 0.05). In contrast, *Asterarcys* sp. MOJF-1, *Asterarcys* sp. PA2, and *Neochloris* sp. PA3 exhibited comparable scavenging activities of approximately 69%, 65%, and 66%, respectively, with no significant differences among these three isolates.

Differences were also observed in the ability of the microalgal extracts to chelate ferrous ions (Figure 3C). Among the four extracts, *Neochloris* sp. PA3 exhibited detectable ferrous ion-chelating activity, reaching approximately 31.5% at 1 mg/mL. In contrast, no detectable chelating activity was observed for *Asterarcys* sp. MOJF-1, *Asterarcys* sp. PA2, or *Chlorella* sp. PS under the experimental conditions. EDTA, used as the positive control, exhibited approximately 86.6% ferrous ion-chelating activity.

Overall, the antioxidant profiles differed according to both the microalgal isolate and the antioxidant mechanism evaluated. Whereas *Chlorella* sp. PS showed the strongest superoxide radical scavenging activity, *Neochloris* sp. PA3 exhibited the highest total antioxidant capacity and was the only extract displaying detectable ferrous ion-chelating activity under the conditions tested. These findings indicate that the antioxidant potential of the microalgal extracts involves distinct and complementary mechanisms.

### 2.3. Untargeted Metabolomic Profiling of Neochloris sp. PA3

Untargeted LC-HRESI-MS/MS profiling of the methanolic extract of *Neochloris* sp. PA3 revealed a chemically diverse metabolomic profile. A total of 227 features associated with MS^2^ spectra were detected, including 181 features in positive ionization mode and 46 in negative ionization mode. Spectral-library matching and in silico annotation resulted in 35 annotated metabolites at MSI levels 2 and 3 (Table S4). Molecular networking further revealed the organization of these features into several chemically related clusters (Figure 4A). Representative subnetworks highlighting selected annotated metabolites, are shown in Figure 4B.

The annotated metabolome was dominated by lipid-related metabolites, particularly fatty acids and fatty-acid derivatives, together with glycerolipids, phospholipids, and apocarotenoid-related metabolites. Among the annotated compounds were loliolide, dihydroactinidiolide, pinolenic acid, palmitoleic acid, trans-10,cis-12-octadecadienoic acid, and 1-monolinolein (Table S4). These results provide a preliminary chemical characterization of the biologically active PA3 methanolic extract and reveal a chemically diverse mixture containing several metabolite classes previously associated with biological activities (Figure 4B).

### 2.4. Cytotoxic Activity Screening Identifies Neochloris sp. PA3 as the Most Active Microalgal Extract

The antiproliferative activity of methanolic extracts obtained from the four microalgal isolates was initially evaluated against five human cancer cell lines, including breast (MCF7), colon (Caco2), prostate (DU145), leukemia (MOLT-4), and ovarian (OVCAR-3), using the MTT assay. Two clinically used chemotherapeutic agents, doxorubicin (DOX) and paclitaxel (PTX), were included as reference compounds.

As shown in Table 1, all four microalgal extracts exhibited cytotoxic activity; however, their potency differed markedly among cell lines. Among the evaluated extracts, *Neochloris* sp. PA3 consistently exhibited the broadest antiproliferative activity, displaying IC_50_ values of 17.1 ± 1.6, 19.0 ± 0.9, and 19.1 ± 1.2 μg/mL against MCF7, Caco2, and DU145 cells, respectively. According to the cytotoxicity criteria proposed by the U.S. National Cancer Institute (NCI), crude extracts with IC_50_ values below 20 μg/mL are considered active [21]. Therefore, the methanolic extract of *Neochloris* sp. PA3 was classified as an active extract against three independent solid tumor cell lines.

The remaining isolates exhibited lower or more restricted antiproliferative activity. *Asterarcys* sp. PA2 showed the greatest activity against DU145 cells (IC_50_ = 14.5 ± 1.5 μg/mL), whereas *Chlorella* sp. PS displayed moderate and cell-line-dependent cytotoxicity. In contrast, *Asterarcys* sp. MOJF-1 exhibited only moderate activity against Caco2 and DU145 cells. None of the evaluated extracts exhibited high cytotoxic activity against the ovarian cancer cell line OVCAR-3, which displayed the highest resistance to all four microalgal extracts.

Importantly, the methanolic extract of *Neochloris* sp. PA3 exhibited substantially higher IC_50_ values in the non-tumorigenic cell models Vero (170.9 ± 11.5 μg/mL) and Detroit 548 (192.1 ± 13.9 μg/mL), indicating markedly lower cytotoxicity in the tested non-tumorigenic models than in the most sensitive cancer cell lines. This favorable cytotoxic profile, together with its broad antiproliferative activity against multiple human cancer cell lines, identified *Neochloris* sp. PA3 as the most promising isolate for subsequent mechanistic studies. Moreover, these findings support the continued investigation of freshwater microalgae from oligotrophic desert environments as sources of bioactive natural products exhibiting in vitro cytotoxic activity.

### 2.5. Neochloris sp. PA3 Exhibits Differential Cytotoxicity Between Cancer and Non-Tumorigenic Cell Models

To evaluate whether the cytotoxic activity of *Neochloris* sp. PA3 differed between cancer and non-tumorigenic cell models, the Selectivity Index (SI) was calculated using the IC_50_ values obtained in Vero and Detroit 548 cells relative to each cancer cell line (Table 2). The methanolic extract of *Neochloris* sp. PA3 showed the greatest differential cytotoxicity for MCF7, Caco2, and DU145 cells, with SI values ranging from approximately 9 to 11 when Detroit 548 cells were used as the reference model and from 8 to 10 using Vero cells. These values greatly exceeded the threshold of SI > 2, which is generally considered indicative of preferential cytotoxicity [47,48]. Moderate differential cytotoxicity was observed for MOLT-4 cells, whereas little or no preferential activity was observed for OVCAR-3 cells.

Although comparable SI values were obtained using the two non-tumorigenic reference cell lines, these findings should be interpreted within the limitations of the cellular models employed. Vero cells are derived from African green monkey kidney and therefore do not represent a normal human cell model, whereas Detroit 548 represents a single human fibroblast-type cell model. Accordingly, the present results demonstrate differential cytotoxicity between the tested cancer and non-tumorigenic cell lines but do not establish general selectivity toward normal human cells or tissues. Additional studies using a broader panel of non-tumorigenic human cell types will be required to determine the extent and tissue specificity of this differential cytotoxicity.

### 2.6. Time-Dependent Antiproliferative Activity of Neochloris sp. PA3

To investigate the biological activity of *Neochloris* sp. PA3, the time-dependent effect of its methanolic extract was evaluated in the three most sensitive cancer cell lines (MCF7, Caco2, and DU145), together with the non-tumorigenic Detroit 548 cell line. Cells were treated with the corresponding IC_50_ concentration of the extract, and cell viability was monitored for up to 60 h using the MTT assay.

As shown in Figure 5, the methanolic extract induced a progressive reduction in cell viability in all three cancer cell lines, whereas Detroit 548 cells were considerably less affected throughout the experimental period. Interestingly, Detroit 548 cells exhibited a slight increase in metabolic activity during the first 24 h of treatment, followed by a gradual decline that remained above 75% viability at the end of the experiment.

Among the cancer cell lines, MCF7 exhibited the earliest and most pronounced response to treatment. Cell viability decreased significantly within the first 12 h and continued to decline throughout the experiment, reaching approximately 23% after 60 h of exposure. A similar time-dependent pattern was observed in Caco2 cells, although the reduction in viability became more pronounced after 48 h, ultimately reaching approximately 25% at 60 h. In contrast, DU145 cells displayed a more moderate response, with viability decreasing to approximately 55% after 24 h and remaining relatively stable during the subsequent time points.

Overall, these findings demonstrate that the antiproliferative activity of the methanolic extract of *Neochloris* sp. PA3 is time-dependent and varies according to the cellular model. The sustained reduction in viability observed in MCF7 and Caco2 cells, together with the limited effect on non-tumorigenic Detroit 548 cells, further supports the differential cytotoxicity of this microalgal extract across the tested cell models and provides a rationale for investigating apoptosis-associated cellular events in subsequent experiments.

### 2.7. Neochloris sp. PA3 Induces Morphological Alterations Consistent with Apoptotic Cell Death

Morphological alterations induced by the methanolic extract of *Neochloris* sp. PA3 were evaluated in MCF7, Caco2, DU145, and the non-tumorigenic Detroit 548 cell line during the first 12 h of treatment. Untreated cells (negative control) maintained their characteristic morphology and remained firmly attached to the culture surface throughout the observation period (Figure 6).

In contrast, treatment with the PA3 extract induced progressive morphological alterations in all three cancer cell lines. As early as 8 h after treatment, MCF7, Caco2, and DU145 cells exhibited evident cell rounding, progressive loss of adherence, reduction in cell spreading, and increased cellular refringence, all of which are morphological features commonly associated with apoptotic cell death. These alterations became more evident after 12 h, when numerous rounded and partially detached cells were observed. Similar morphological changes were detected in cells treated with doxorubicin (DOX), the positive control, confirming that the response induced by the extract resembled that produced by a clinically established chemotherapeutic agent.

In contrast, Detroit 548 cells treated with the methanolic extract largely preserved their normal fibroblast-like morphology and remained attached to the culture surface throughout the experiment, exhibiting only minor morphological alterations compared with untreated controls. Conversely, DOX treatment produced marked cell rounding and extensive loss of adhesion in Detroit 548 cells after 12 h of exposure.

These observations are consistent with the cytotoxicity analysis and show that *Neochloris* sp. PA3 treatment produced more pronounced apoptosis-associated morphological alterations in the cancer cell lines than in Detroit 548 cells.

### 2.8. AO/EB Staining Reveals Differential Changes in Cell Viability and Nuclear Morphology Induced by Neochloris sp. PA3

Changes in cell viability and nuclear morphology induced by the methanolic extract of *Neochloris* sp. PA3 were investigated using dual acridine orange/ethidium bromide (AO/EB) staining after 4 h of treatment. The assay was performed in MCF7, Caco2, DU145, and the non-tumorigenic Detroit 548 cell line, with doxorubicin (DOX) and paclitaxel (PTX) included as reference anticancer drugs. Representative fluorescence micrographs (Figure 7) showed that untreated cells remained predominantly viable, exhibiting uniformly green nuclei with normal nuclear morphology. In contrast, treatment with the methanolic extract of *Neochloris* sp. PA3 produced evident nuclear alterations in all three cancer cell lines, including chromatin condensation, nuclear fragmentation, and the appearance of yellow-green and orange fluorescent nuclei. These morphological features are consistent with early and late stages of apoptotic cell death, respectively, and resembled those observed in cells treated with DOX. In comparison, PTX treatment produced a greater proportion of red fluorescent nuclei, indicating a higher number of non-viable cells.

Quantitative analysis of at least 300 cells per treatment confirmed these observations (Figure 8). Exposure to the PA3 extract significantly reduced the proportion of viable cells in MCF7, Caco2, and DU145 cultures while increasing the percentage of cells exhibiting nuclear features compatible with early and late apoptosis. In MCF7 and DU145 cells, most non-viable cells displayed apoptotic nuclear morphology, whereas Caco2 cells showed a comparatively larger proportion of non-viable cells. Importantly, Detroit 548 cells remained predominantly viable after treatment with the methanolic extract, whereas DOX and particularly PTX markedly increased the proportion of non-viable cells.

Overall, AO/EB staining demonstrated that the methanolic extract of *Neochloris* sp. PA3 selectively reduces the viability of human cancer cells while preserving the viability of non-tumorigenic cells. Furthermore, the nuclear alterations observed following treatment are consistent with apoptotic cell death, although this assay alone cannot establish apoptosis as the definitive mechanism of cell death. Therefore, the involvement of executioner caspase activity in the cellular response was subsequently evaluated using a caspase-3/7 assay.

### 2.9. Neochloris sp. PA3 Treatment Increases Executioner Caspase-3/7 Activity in Human Cancer Cells

Caspase-3/7 activity was evaluated to determine whether the reduction in cell viability and the nuclear alterations observed following treatment with the methanolic extract of *Neochloris* sp. PA3 were associated with increased executioner caspase activity. Caspase-3/7 activity was analyzed after 4 h of treatment in MCF7, Caco2, DU145, and the non-tumorigenic Detroit 548 cell line using the CellEvent™ Caspase-3/7 Green Detection Reagent. Doxorubicin (DOX) was included as the positive control.

Representative fluorescence micrographs (Figure 9) revealed virtually no detectable caspase-3/7-associated fluorescence in untreated cells. In contrast, treatment with the methanolic extract of *Neochloris* sp. PA3 produced a marked increase in green fluorescent cells in all three cancer cell lines, indicating increased executioner caspase-3/7 activity. This response was most pronounced in MCF7 cells, followed by Caco2 and DU145 cells, whereas only occasional fluorescent cells were detected in Detroit 548 cultures. DOX also increased caspase-3/7 activity in cancer cells; however, fewer fluorescent cells were observed than in the corresponding PA3-treated cultures under the experimental conditions evaluated.

Quantitative analysis confirmed these observations (Figure 10). Treatment with the methanolic extract of *Neochloris* sp. PA3 significantly increased the percentage of caspase-3/7-positive cells in all three cancer cell lines compared with untreated controls (*p* < 0.05). MCF7 cells exhibited the highest percentage of caspase-3/7-positive cells (approximately 26%), followed by Caco2 (18%) and DU145 (13%). In contrast, Detroit 548 cells displayed only minimal caspase-3/7-associated fluorescence (approximately 0.5%). Although DOX also significantly increased caspase-3/7 activity, the percentage of positive cells remained consistently lower than that observed following PA3 treatment in all three cancer cell lines at the analyzed time point.

Importantly, these findings are consistent with the progressive loss of cell viability, morphological alterations, and AO/EB staining patterns observed in the previous experiments and provide additional evidence of an apoptosis-associated cellular response involving increased executioner caspase activity. Because the CellEvent™ assay detects combined caspase-3/7 activity and does not distinguish between the individual enzymes, the observed fluorescence cannot be attributed specifically to either caspase-3 or caspase-7. This distinction is particularly relevant for MCF7 cells, which are known to lack functional caspase-3 expression. Therefore, the comparatively high CellEvent™ signal observed in PA3-treated MCF7 cells should be interpreted as increased executioner caspase activity rather than specific caspase-3 activation. Overall, the markedly greater caspase-3/7-associated response observed in the cancer cell lines compared with Detroit 548 cells supports an apoptosis-associated effect of the PA3 extract involving executioner caspase activity, although these findings do not establish a specific caspase-dependent apoptotic pathway.

## 3. Discussion

Microalgae are increasingly recognized as promising reservoirs of structurally and functionally diverse metabolites with potential value for cancer-related biomedical research. However, most investigations have focused on marine species or on a relatively small number of extensively studied genera, whereas freshwater microalgae inhabiting extreme or oligotrophic environments remain comparatively underexplored. In the present study, four chlorophyte strains isolated from freshwater systems within the Cuatro Ciénegas Basin were identified as *Asterarcys* sp. MOJF-1, *Asterarcys* sp. PA2, *Neochloris* sp. PA3, and *Chlorella* sp. PS. Among these isolates, *Neochloris* sp. PA3 emerged as the most promising strain based on the combination of antioxidant activity, broad antiproliferative effects, differential cytotoxicity across the tested cell models, and an apoptosis-associated cellular response involving increased executioner caspase-3/7 activity. These findings identify microalgae isolated from the oligotrophic environments of the Cuatro Ciénegas Basin as an underexplored source of biologically active natural products; however, they do not establish that oligotrophic adaptation is responsible for the observed biological activities.

The biological potential of microalgae is strongly influenced by their taxonomic identity, environmental origin, cultivation conditions, and metabolic composition. Microalgae synthesize carotenoids, polyphenols, fatty acids, sterols, polysaccharides, peptides, and other secondary metabolites capable of exerting antioxidant and anticancer effects through multiple cellular mechanisms [49,50]. Recent evidence also indicates that cultivation conditions can markedly modify the biochemical composition and antioxidant properties of microalgae, including *Neochloris oleoabundans* [51]. This is particularly relevant for organisms originating from the Cuatro Ciénegas Basin, an ecosystem characterized by extreme oligotrophy and unusual nutrient stoichiometry. Environmental pressures associated with oligotrophic habitats could contribute to distinctive metabolic profiles; nevertheless, the present study did not experimentally evaluate the effects of nutrient limitation or other environmental stressors on metabolite production, and therefore a causal relationship between oligotrophic adaptation and the observed biological activities cannot be established.

The antioxidant analyses revealed substantial differences among the four microalgal extracts, with antioxidant performance varying according to the mechanism evaluated. *Neochloris* sp. PA3 exhibited the highest total antioxidant capacity and was the only extract showing detectable ferrous ion-chelating activity under the experimental conditions, whereas *Chlorella* sp. PS displayed the strongest superoxide radical scavenging activity. These differences are consistent with the chemically heterogeneous nature of microalgal extracts, in which distinct classes of metabolites may contribute to antioxidant effects through complementary mechanisms, including radical scavenging, electron transfer, and metal chelation. Recent comparative analyses have similarly demonstrated marked interspecies and extraction-dependent variation in the antioxidant capacity of microalgae [52]. Collectively, these findings emphasize that antioxidant potential cannot be adequately characterized by a single assay and highlight the value of integrating complementary methods to obtain a more comprehensive assessment of the antioxidant profile of microalgal extracts.

The coexistence of antioxidant and anticancer activities in the PA3 extract is particularly interesting because these properties should not be regarded as biologically contradictory. Microalgal metabolites may exert antioxidant effects in cell-free chemical assays while simultaneously disrupting redox homeostasis, mitochondrial function, survival signaling, or other processes in cancer cells [49]. The biological outcome of a complex extract ultimately depends on its composition, concentration, cellular uptake, and the metabolic characteristics of the target cell. Consequently, the antioxidant activity demonstrated here should be considered an additional biological property of the PA3 extract rather than evidence that its cytotoxicity necessarily results from suppression of oxidative stress. Determination of intracellular reactive oxygen species and mitochondrial function after PA3 exposure will be required to establish whether redox modulation contributes directly to its anticancer mechanism.

A major finding of this study was the pronounced difference in cytotoxic potency among the four isolates. *Neochloris* sp. PA3 exhibited IC_50_ values of 17.1, 19.0, and 19.1 μg/mL against MCF7, Caco2, and DU145 cells, respectively, indicating substantial activity against three tumor models of different tissue origin. In contrast, OVCAR-3 cells were markedly resistant to all four microalgal extracts, demonstrating that the response is strongly dependent on cellular context. Such heterogeneity is expected because tumor cell lines differ substantially in their genetic background, metabolic dependencies, membrane transport systems, apoptotic competence, and resistance mechanisms. The differential responses observed here therefore suggest that the active constituents of PA3 may interact with molecular vulnerabilities that are present in breast, colorectal, and prostate cancer cells but less prominent in the ovarian cancer model evaluated.

Evidence specifically involving the genus *Neochloris* is particularly relevant to the present findings. Castro-Puyana et al. demonstrated that carotenoid-rich extracts obtained from *Neochloris oleoabundans* inhibited proliferation of human colorectal cancer cells, including HT-29, SW480, and a primary colon cancer-derived cell line [53]. Their chemical analysis identified lutein and carotenoid esters among the major constituents and revealed associations between carotenoid composition and antiproliferative activity. More recently, *N. oleoabundans* has been shown to possess a metabolically diverse composition and antioxidant properties that vary substantially according to cultivation conditions [51]. Although the species and extraction procedures differ from those used in the present study, these observations provide independent evidence that members of the genus *Neochloris* can produce metabolites with both antioxidant and antiproliferative potential. The present results extend these observations by demonstrating activity of a *Neochloris* isolate against breast, colon, and prostate cancer cells and, importantly, substantially lower cytotoxicity toward the non-tumorigenic reference cell lines evaluated in this study.

The IC_50_ values of the PA3 extract against Vero and Detroit 548 cells were approximately 171 and 192 μg/mL, respectively, almost one order of magnitude higher than those observed in the most sensitive cancer cell lines. Consequently, SI values of approximately 9–11 were obtained for MCF7, Caco2, and DU145 cells, depending on the reference cell line. The comparable SI values obtained using two different non-tumorigenic cell lines strengthen the evidence that the observed differential cytotoxicity was not restricted to a single reference model. Recent studies have likewise emphasized the importance of comparing the cytotoxic effects of microalgal preparations across cancer and non-tumorigenic cell models. For example, a recent investigation of *Chlorella vulgaris* BA02 reported differential cytotoxic responses among breast cancer cell lines and demonstrated that the biological activity of microalgal extracts can be highly dependent on cellular phenotype [47]. Thus, validation using a broader panel of non-tumorigenic human cells from different tissues will be necessary to determine whether the differential cytotoxicity observed here extends across additional cellular models.

The time-course experiments further demonstrated that the effects of PA3 were dynamic and cell-line dependent. MCF7 cells responded rapidly, showing a progressive reduction in metabolic viability that reached approximately 23% after 60 h, whereas Caco2 cells displayed a somewhat delayed but ultimately comparable response. DU145 cells exhibited a more moderate decrease that stabilized after approximately 24–36 h. In contrast, Detroit 548 cells retained more than 75% viability after 60 h. These results reinforce the differential citotoxicity observed in the 24-h IC_50_ experiments and indicate that prolonged exposure does not eliminate the differential response between cancer and non-tumorigenic cells. The distinct kinetics among the cancer models may reflect differences in uptake or metabolism of extract components, basal proliferative rates, or differential engagement of cell-death pathways.

Microscopic observations were consistent with the viability assays. PA3-treated MCF7, Caco2, and DU145 cells progressively developed rounding, reduced spreading, increased refringence, and loss of adherence, whereas Detroit 548 fibroblasts largely maintained their characteristic morphology. These changes resemble morphological events commonly associated with apoptotic cell death. Similar effects have previously been reported for microalgal extracts. Methanolic extracts of *Chlorella sorokiniana* and *Scenedesmus* sp. isolated in northern Mexico were shown to exert antitumor activity accompanied by morphological and biochemical evidence of apoptosis [54]. Likewise, *Chlorella* extracts have been reported to induce apoptotic responses in several human cancer models, indicating that programmed cell death may represent a recurrent biological response to selected microalgal metabolites [48,49].

The AO/EB results provide additional evidence of PA3-induced loss of cancer-cell viability and nuclear alterations compatible with apoptosis. PA3 treatment increased the proportion of cells displaying yellow-green or orange nuclei, chromatin condensation, and nuclear fragmentation in MCF7, Caco2, and DU145 cultures, while Detroit 548 cells remained predominantly viable. However, AO/EB staining is fundamentally a viability and morphological discrimination method and is not sufficient by itself to establish apoptosis as the mechanism of cell death. Accordingly, these observations should be interpreted as evidence of membrane and nuclear changes consistent with apoptotic progression rather than definitive proof of apoptosis. This distinction is important because several forms of regulated and non-regulated cell death can share morphological features.

Treatment with the *Neochloris* sp. PA3 extract also increased executioner caspase-3/7 activity in the three cancer cell lines, whereas only minimal activity was detected in Detroit 548 cells. The response was particularly pronounced in MCF7 cells, in which approximately 26% of cells were positive for the CellEvent™ Caspase-3/7 signal, 18% in Caco2, and 13% in DU145 after 4 h. However, interpretation of this finding requires consideration of the well-established caspase-3 deficiency of MCF7 cells. Jänicke et al. demonstrated that MCF7 cells lack functional caspase-3 as a consequence of a 47-base-pair deletion within the *CASP3* gene that results in aberrant pre-mRNA splicing and abrogates translation of caspase-3 [55]. Therefore, because the CellEvent™ Caspase-3/7 assay detects the activity of both executioner caspases without distinguishing between them, the fluorescence observed in PA3-treated MCF7 cells cannot be attributed to caspase-3 activation and likely predominantly reflects caspase-7 activity. More generally, the assay demonstrates increased executioner caspase-3/7 activity in PA3-treated cancer cells but does not identify the individual caspase responsible for the signal. Taken together with the morphological alterations and AO/EB staining patterns, these findings support an apoptosis-associated cellular response involving executioner caspase activity. Nevertheless, the present data do not establish a specific caspase-dependent apoptotic pathway, and additional analyses of individual caspases and other apoptotic markers would be required to define the underlying mechanism.

These findings agree with recent mechanistic studies of other green microalgae. Sawasdee et al. demonstrated that an ethanolic *Chlorella* sp. extract induced apoptosis in cholangiocarcinoma cells, accompanied by increased caspase activity, reduced Bcl-2 expression, and inhibition of AKT/mTOR survival signaling [48]. Microalgal and cyanobacterial metabolites have also been reported to modulate mitochondrial function, oxidative stress, protein kinases, cell-cycle progression, and apoptosis-related signaling pathways [49]. The present study does not establish which upstream pathways contribute to the caspase-3/7 response following PA3 exposure, but the rapid activity detected after 4 h suggests that early intracellular signaling events precede the more pronounced reductions in viability observed at later time points.

Interestingly, PA3 produced a higher proportion of caspase-3/7-positive cancer cells than DOX at the specific 4-h time point evaluated. This finding should not be interpreted as evidence that the extract is more potent or therapeutically superior to doxorubicin. The two treatments were tested at their respective IC_50_ concentrations, and the kinetics of executioner caspase activity can differ considerably among compounds and cellular models. Therefore, this result indicates only that PA3 produced a greater detectable caspase-3/7-associated response under the specific experimental conditions and at the time point analyzed. Detailed time-course studies of caspase activity, together with measurements of initiator caspases, mitochondrial membrane potential, cytochrome *c* release, BAX/BCL-2 balance, and PARP cleavage, will be necessary to determine whether specific apoptosis pathways contribute to the cytotoxic effects of PA3.

The chemical identity of the molecules responsible for these effects remains an important unresolved question. Microalgae contain numerous potentially bioactive molecules, including carotenoids, chlorophyll derivatives, sterols, polyunsaturated fatty acids, phenolic compounds, polysaccharides, and peptides [49,50]. Carotenoids deserve particular attention because previous work with *N. oleoabundans* associated its antiproliferative activity with carotenoid-rich fractions [53].

The untargeted LC-HRESI-MS/MS analysis substantially expanded the chemical characterization of the PA3 extract and revealed a metabolically diverse mixture dominated by lipid-related compounds, together with apocarotenoid derivatives and other metabolite classes. Several tentatively annotated metabolites are noteworthy because related biological activities have previously been reported. Interestingly, several metabolites tentatively annotated in the *Neochloris* sp. PA3 extract have previously been associated with cytotoxic or anticancer-related activities. Among these, trans-10,cis-12-octadecadienoic acid, corresponding to the trans-10,cis-12 isomer of conjugated linoleic acid (t10,c12-CLA), is particularly noteworthy. This CLA isomer has been shown to inhibit the growth of human Caco-2 colon cancer cells by decreasing DNA synthesis and inducing apoptosis, whereas the cis-9,trans-11 isomer showed substantially lower activity [56]. Similar pro-apoptotic effects of t10,c12-CLA have been reported in HT-29 colon cancer cells, involving mitochondrial membrane depolarization, cytochrome c release, and activity of executioner caspases [57]. These observations are of particular interest because Caco2 was among the cancer cell lines most sensitive to the PA3 extract in the present study.

Other annotated metabolites may provide additional chemical context for the observed biological activity. 1-Monolinolein has previously exhibited cytotoxic activity against human A549 lung adenocarcinoma cells, with a reported IC_50_ of 3.6 μM [58]. Loliolide is also noteworthy because this monoterpene lactone, isolated from the marine microalga *Tisochrysis lutea*, significantly reduced the viability of HepG2 hepatocellular carcinoma cells while displaying lower toxicity toward a non-tumorigenic cell model [59]. Dihydroactinidiolide, another apocarotenoid lactone annotated in the PA3 extract, has also been investigated in cancer-related models, although the evidence supporting direct cytotoxic activity is less extensive. Collectively, the occurrence of metabolites with previously reported anticancer-related properties provides plausible chemical context for the biological activity observed for the PA3 extract. Although individual metabolites identified in the PA3 extract have previously been associated with cytotoxic activity, no causal relationship can presently be established between any of these metabolites and the cytotoxic or apoptosis-associated effects observed in this study. Confirmation through bioassay-guided fractionation of the PA3 extract and individual evaluation of the isolated compounds in the cancer cell lines tested in this study will be required to identify the constituents responsible for the observed activity.

Although the metabolomic characterization provides chemical context for the biological activity of the PA3 extract, the present analysis was restricted to PA3 and therefore does not establish whether the annotated metabolites are unique to this strain. The differential cytotoxicity observed among the four microalgal extracts could reflect qualitative differences in metabolite composition, differences in the relative abundance of shared metabolites, or differences in extraction efficiency. Comparative metabolomic profiling of all four extracts, together with standardized extraction-yield determination and quantitative analysis of selected metabolites, will therefore be required to identify the chemical features specifically associated with the greater cytotoxic activity of PA3.

An additional aspect that distinguishes the present study is the ecological origin of the isolates. The Cuatro Ciénegas Basin represents an unusual desert oasis characterized by long-term nutrient limitation and distinctive microbial communities. Extremotolerant and extremophilic microalgae are increasingly being explored as sources of valuable metabolites because adaptation to environmental stress can substantially reshape cellular metabolism and the production of protective molecules [11]. Nevertheless, a direct causal relationship between the oligotrophic environment and the anticancer properties of PA3 remains hypothetical. Comparative metabolomic studies involving PA3 cultured under different phosphorus, nitrogen, irradiance, and oxidative-stress conditions would be particularly informative for determining whether environmental stress modulates the production of the compounds responsible for its biological activity.

Taken together, the findings demonstrate that freshwater microalgae isolated from the oligotrophic ecosystems of the Cuatro Ciénegas Basin constitute an insufficiently explored source of biologically active natural products, without establishing a causal relationship between their environmental origin and the observed biological activities. Among the four isolates investigated, *Neochloris* sp. PA3 showed a particularly favorable profile by combining antioxidant properties with potent cytotoxicity toward several human cancer cell lines together with substantially lower cytotoxicity in the tested non-tumorigenic cell models and executioner caspase-3/7 activity. Nevertheless, the present results represent an initial in vitro characterization rather than evidence of therapeutic efficacy. Future studies should prioritize bioassay-guided fractionation of the PA3 methanolic extract followed by LC–MS/MS and complementary metabolomic analyses to identify the compounds responsible for its activity. The most active fractions and purified compounds should subsequently be evaluated for intracellular ROS modulation, mitochondrial dysfunction, cell-cycle alterations, and intrinsic and extrinsic apoptotic signaling. Validation in three-dimensional tumor spheroids and, subsequently, appropriate in vivo models will also be essential to determine whether the selectivity observed in monolayer cultures is maintained in more physiologically relevant systems. Such studies may establish whether *Neochloris* sp. PA3 represents not only an ecologically distinctive microalga but also a source of bioactive molecules warranting further preclinical investigation.

## 4. Materials and Methods

### 4.1. Collection and Isolation of Microalgal Strains

Microalgal samples were collected from three freshwater hydrological systems, Mojarral (26°54′06″ N, 102°11′10″ W), Poza Azul, and Poza Sal (26°49′39.03″ N, 102°01′26.94″ W), located within the Cuatro Ciénegas Basin, Coahuila, Mexico, between February and July 2016. Sampling was conducted under collection permit No. SGPA/DGVS/03121/15 issued by the Secretaría de Medio Ambiente y Recursos Naturales (SEMARNAT, Mexico).

Freshwater samples were thoroughly mixed under sterile conditions, and aliquots were spread onto Petri dishes containing BG-11 medium (Cat. No. B1511, PhytoTech Labs, Lenexa, KS, USA) supplemented with 1.5% (*w*/*v*) bacteriological agar, following a standard protocol for freshwater microalgae isolation [60]. Pure cultures were obtained by repeated subculturing onto fresh BG-11 agar plates.

The purified isolates were designated MOJF-1 (Mojarral #1), PA2 (Poza Azul #2), PA3 (Poza Azul #3), and PS (Poza Sal), and subsequently maintained in liquid BG-11 medium for routine cultivation and biomass production. Cultures were incubated at 22–25 °C under a 16:8 h light/dark photoperiod, 70% relative humidity, and a photosynthetic photon flux density of 100 µmol photons m^−2^ s^−1^.

Cell morphology was routinely examined using a B-292 bright-field microscope (OPTIKA, Ponteranica, Bergamo, Italy).

### 4.2. Molecular Identification of Microalgal Strains

Genomic DNA was extracted from each microalgal isolate using a phenol/chloroform extraction protocol followed by DNA precipitation with sodium chloride (NaCl) and cold ethanol. The extracted DNA was used as template for amplification of the chloroplast *rbcL* gene using the primer pair rbcL-192F (5′-GGTACTTGGACAACWGTTWTGGAC-3′) and rbcL-657R (5′-GAAACGGTCTCKCCARCGCAT-3′). The *rbcL* gene, which encodes the large subunit of ribulose-1,5-bisphosphate carboxylase/oxygenase (RuBisCO), is widely used as a molecular marker for phylogenetic identification of green microalgae. In the present study, partial *rbcL* sequences were used primarily to support genus-level taxonomic assignment. Because partial *rbcL* sequences may not provide sufficient resolution for unequivocal discrimination among closely related species, species-level assignments were not attempted.

PCR amplification was carried out in a final reaction volume of 25 μL using DreamTaq™ PCR Master Mix (2×) (Cat. No. K1071, Thermo Fisher Scientific, Vilnius, Lithuania) and primers at a final concentration of 1 μM each. Reactions were performed in a TC1000-G thermal cycler (DLAB Scientific Co., Ltd., Beijing, China) under the following cycling conditions: an initial denaturation at 95 °C for 1 min; 35 amplification cycles consisting of denaturation at 95 °C for 30 s, primer annealing at 58 °C for 30 s, and extension at 72 °C for 1 min; followed by a final extension at 72 °C for 5 min.

PCR products were analyzed by electrophoresis on 1% (*w*/*v*) agarose gels stained with RedGel™ nucleic acid stain (Biotium Inc., Fremont, CA, USA) and visualized under ultraviolet illumination. The amplified *rbcL* fragments were subsequently cloned into the pCR™4-TOPO^®^ vector (Invitrogen, Carlsbad, CA, USA) according to the manufacturer’s instructions. Recombinant plasmids containing the *rbcL* amplicons were commercially sequenced by the Laboratorio Nacional de Biotecnología Agrícola, Médica y Ambiental (LANBAMA), Instituto Potosino de Investigación Científica y Tecnológica (IPICYT), San Luis Potosí, Mexico.

### 4.3. Phylogenetic Reconstruction

The obtained *rbcL* nucleotide sequences were compared against the National Center for Biotechnology Information (NCBI) database using the Basic Local Alignment Search Tool (BLASTn ver 2.17.0) to identify the closest phylogenetic relatives and determine the putative taxonomic affiliation of each isolate at the genus level. Based on the BLAST results, representative *rbcL* sequences from related chlorophyte taxa were retrieved from GenBank for phylogenetic analyses. The reference sequences used for the phylogenetic reconstruction of *Asterarcys* sp. MOJF-1 and PA2, *Neochloris* sp. PA3, and *Chlorella* sp. PS are provided in Table S1, Table S2 and Table S3, respectively [15,16,17,18,19,20,21,22,23,24,25,26,27,28,29,30,31,32,33,34,35,36,37,38,39,40,41,42,43,44,45,46]. The phylogenetic trees were rooted using *Bracteacoccus aerius* (Bracteacoccaceae) or *Pseudococcomyxa simplex* (Oocystaceae), as appropriate for the taxonomic group analyzed; the specific outgroup used for each phylogenetic reconstruction is indicated in the corresponding figure legend.

Sequence alignment was performed using the MUSCLE algorithm with the default parameters [61], as implemented in MEGA version 11 [62]. The alignments were manually inspected and trimmed to a common overlapping region prior to phylogenetic reconstruction. Maximum Likelihood (ML) phylogenetic trees were reconstructed in MEGA11 using the best-fit nucleotide substitution model selected with the Find Best DNA/Protein Models (ML) function implemented in the software. The General Time Reversible model with gamma-distributed rate variation among sites (GTR + G) was selected as the optimal evolutionary model for the analyzed dataset. The robustness of the inferred phylogenies was evaluated by bootstrap analysis using 1000 pseudoreplicates [63].

The partial *rbcL* nucleotide sequences corresponding to *Neochloris* sp. PA3, *Asterarcys* sp. MOJF-1, *Asterarcys* sp. PA2, and *Chlorella* sp. PS were deposited in the NCBI database under accession numbers PZ759594, PZ759593, PZ776196, and PZ759595, respectively.

### 4.4. Preparation of Microalgal Extracts

For biomass production, each isolate was initially cultured in 200 mL of liquid BG-11 medium and subsequently scaled up by transferring the culture into 800 mL of fresh BG-11 medium (Cat. No. B1511, PhytoTech Labs, Lenexa, KS, USA). Cultures were incubated at 22–25 °C under a 16:8 h light/dark photoperiod, 70% relative humidity, and a photosynthetic photon flux density of 100 µmol photons m^−2^ s^−1^ for 7 days.

Following cultivation, the microalgal biomass was harvested by centrifugation at 4000 rpm for 15 min at 4 °C using an Eppendorf Centrifuge 5810 R (Eppendorf AG, Hamburg, Germany). The resulting wet biomass was extracted with methanol (MeOH) (Cat. No. 06125, Fermont, Monterrey, Nuevo León, Mexico) at a 1:1 (*w*/*v*) biomass-to-solvent ratio under continuous agitation (200 rpm) in the dark for 5 days. The methanolic extracts were subsequently filtered through Whatman No. 1 filter paper to remove cellular debris, and the solvent was evaporated to dryness in a convection incubator at 30 °C to obtain the crude extracts. Methanol used during extraction was evaporated to dryness before preparation of the stock solutions; therefore, methanol was not intentionally present in the solutions used for cell-based assays. To minimize chemical degradation, dried crude extracts were stored at −20 °C in amber tubes protected from light until analysis. Following solvent evaporation, the dried crude extracts were weighed and the extraction yield was calculated as the percentage of crude extract recovered relative to the initial wet biomass used for extraction. The extraction yields were 0.8%, 0.75%, 1%, and 0.93% for *Asterarcys* sp. MOJF-1, *Asterarcys* sp. PA2, *Neochloris* sp. PA3, and *Chlorella* sp. PS, respectively.

For cytotoxicity assays, the dried crude extracts were dissolved in dimethyl sulfoxide (DMSO) (Cat. No. D128-1, Fisher Chemical, Fair Lawn, NJ, USA) to obtain a stock solution of 100 mg/mL. Working solutions were freshly prepared by diluting the stock solution in sterile 1× phosphate-buffered saline (PBS) (Cellgro, Manassas, VA, USA; Cat. No. 21-040-CV) immediately before use. The final DMSO concentration in cell culture did not exceed 0.1% (*v*/*v*), and the corresponding vehicle concentration was used in control wells. To minimize potential variability associated with prolonged storage and repeated freeze-thaw cycles, all biological assays were performed using extracts prepared within six months prior to testing. This criterion was established based on preliminary observations from our laboratory indicating that some extracts may exhibit reduced biological activity following extended storage and repeated freeze-thaw cycles.

### 4.5. Untargeted LC-HRESI-MS/MS Metabolomic Analysis

#### 4.5.1. LC-HRESI-MS/MS Analysis

Untargeted metabolomic profiling of the *Neochloris* sp. PA3 methanolic extract was performed by liquid chromatography coupled to high-resolution electrospray ionization tandem mass spectrometry (LC-HRESI-MS/MS), following a previously reported methodology [64] with minor modifications. Chromatographic separation was performed using a Waters ACQUITY UPLC system coupled to a Q Exactive Plus high-resolution mass spectrometer (Thermo Fisher Scientific) equipped with a heated electrospray ionization (HESI) source. Metabolites were separated on an ACQUITY UPLC BEH C18 column (50 × 2.1 mm, 1.7 μm, 130 Å) maintained at 40 °C.

The mobile phase consisted of 0.1% (*v*/*v*) formic acid in water (A) and acetonitrile (B). Chromatographic separation was performed using a linear gradient from 15% to 100% B over 8 min, followed by an isocratic step at 100% B for 1.5 min. The flow rate was 0.3 mL/min and the injection volume was 3 μL. The dried *Neochloris* sp. PA3 methanolic extract was dissolved at 0.2 mg/mL in dioxane:methanol (1:1, *v*/*v*) and filtered through a 0.45 μm PTFE syringe filter prior to injection.

Mass spectrometric data were acquired in both positive and negative ionization modes over an *m*/*z* range of 150–2000 using data-dependent acquisition (DDA) with Top-5 precursor fragmentation. The principal ion-source parameters were as follows: spray voltage, 3.80 kV; capillary temperature, 300 °C; capillary voltage, 5 V; tube-lens offset, 35 V; and sheath/auxiliary gas flow, 30 arbitrary units.

#### 4.5.2. LC-MS/MS Data Processing, Metabolite Annotation, and Molecular Networking

Raw LC-HRESI-MS/MS data files were converted to the open-source mzML format using MSConvert [65] and subsequently processed in MZmine 3 [66] for feature detection, alignment, and blank removal in both positive and negative ionization modes. The resulting MGF and CSV files were submitted to the Global Natural Products Social Molecular Networking platform (GNPS2) [67] for spectral dereplication. Spectral-library matches were considere annotations corresponding to Metabolomics Standards Initiative (MSI) level 2 [68]. Spectral matching was performed using precursor- and fragment-ion mass tolerances of 0.02 Da, a cosine-score threshold of 0.7, and a minimum of six matched fragment peaks. Additional spectral annotations were obtained using MS2Query [69] and classified as MSI level 2.

For features lacking spectral-library matches, putative molecular formula/structure information was obtained using CSI:FingerID [70] and reported as MSI level 3 annotations. Chemical classes were predicted using CANOPUS [71] together with the NPClassifier framework [72]. Molecular networks representing the chemical space of the PA3 extract were generated by cross-ionization comparison using MS2DeepScore spectral similarity [73], applying a similarity cutoff of 0.85.

Chemical-class annotations were integrated with the MS2DeepScore molecular network using a modified in-house R workflow adapted from MolNetEnhancer [74]. The resulting molecular network was visualized using Cytoscape version 3.9.0 [75], with nodes colored according to NPClassifier pathway-level assignments and node size scaled according to precursor *m*/*z*. The raw LC-MS/MS datasets generated in positive and negative ionization modes were deposited in the GNPS/MassIVE public repository under accession number MSV000103369.

### 4.6. Antioxidant Activity Assays

To evaluate the antioxidant potential of the methanolic microalgal extracts, three complementary in vitro assays based on different antioxidant mechanisms were performed, including superoxide radical scavenging activity, ferrous ion-chelating activity, and total antioxidant capacity (TAC). The use of multiple assays has been recommended because no single method can accurately reflect the overall antioxidant capacity of complex natural extracts, particularly those derived from microalgae.

#### 4.6.1. Superoxide Radical Scavenging Activity

Superoxide radical scavenging activity was determined with minor modifications [76]. Each 3.0 mL reaction mixture contained 50 mM sodium phosphate buffer (pH 7.8), 13 mM methionine, 100 μM EDTA, 75 μM nitro blue tetrazolium (NBT), 2 μM riboflavin, and methanolic extract (1000 μg/mL). The mixtures were exposed to light for 10 min, and absorbance was measured at 560 nm. Butylated hydroxyanisole (BHA) was used as the positive control. Superoxide scavenging activity was calculated as:

Scavenging activity (%) = [(A_0_ − A_1_)/A_0_] × 100

where A_0_ and A_1_ represent the absorbance of the blank and sample, respectively. Superoxide radical scavenging activity was expressed as the percentage of scavenging activity at an extract concentration of 1000 μg/mL.

#### 4.6.2. Ferrous Ion Chelating Activity

Ferrous ion-chelating activity was determined as previously reported [77], with minor modifications. Briefly, 50 μL of 0.66 mM FeCl_2_ were added to 250 μL of methanolic extract (1000 μg/mL) and incubated at 35 °C with orbital shaking (200 rpm) for 15 min. Subsequently, 200 μL of 1.66 mM ferrozine were added, and the mixtures were incubated for an additional 10 min. Samples were centrifuged at 35,000× *g* for 5 min, and the absorbance of the supernatants was measured at 562 nm. EDTA was used as positive control. Chelating activity was calculated using the following equation:

Chelating activity (%) = [(A_0_ − A_1_)/A_0_] × 100

where A_0_ is the absorbance of the blank and A_1_ is the absorbance of the sample.

#### 4.6.3. Total Antioxidant Capacity

Total antioxidant capacity (TAC) was determined using the phosphomolybdenum assay [78]. Briefly, aliquots of each methanolic extract were mixed with the phosphomolybdenum reagent containing 0.6 M sulfuric acid, 28 mM sodium phosphate, and 4 mM ammonium molybdate. The reaction mixtures were incubated at 95 °C for 90 min, cooled to room temperature, and the absorbance was measured at 695 nm using a UV–Vis spectrophotometer (Thermo Scientific, Waltham, MA, USA). Ascorbic acid was used as the positive control, whereas deionized water replaced the sample in the blank reaction. Results were expressed as μmol ascorbic acid equivalents (AAE) g^−1^ dry weight (DW) according to the ascorbic acid calibration curve. All antioxidant assays were performed in triplicate, and the resulting data were analyzed as described in the Statistical Analysis section.

### 4.7. Cell Lines and Cell Culture

Human breast adenocarcinoma (MCF7, HTB-22), colorectal adenocarcinoma (Caco2, HTB-37), prostate carcinoma (DU145, HTB-81), ovarian adenocarcinoma (OVCAR-3, HTB-161), acute lymphoblastic leukemia (MOLT-4, CRL-1582), African green monkey kidney epithelial (Vero, CCL-81), and human embryonic skin fibroblast (Detroit 548, CCL-116) cell lines were obtained from the American Type Culture Collection (ATCC, Manassas, VA, USA).

MCF7, Caco2, DU145, Vero, and Detroit 548 cells were cultured in high-glucose Dulbecco’s Modified Eagle Medium (DMEM; Gibco, Grand Island, NY, USA; Cat. No. 11995-065) supplemented with 10% (*v*/*v*) fetal bovine serum (FBS; Gibco, Grand Island, NY, USA; Cat. No. 26140-079). OVCAR-3 and MOLT-4 cells were maintained in RPMI-1640 medium (Gibco, Grand Island, NY, USA; Cat. No. 22400-089) supplemented with 10% (*v*/*v*) FBS (Gibco, Grand Island, NY, USA; Cat. No. 26140-079).

All cell lines were maintained at 37 °C in a humidified atmosphere containing 5% CO_2_ and were routinely subcultured at 70–80% confluence before use in the experiments.

### 4.8. Cytotoxicity Assay

The cytotoxic activity of the microalgal extracts was evaluated using the MTT assay [3-(4,5-dimethylthiazol-2-yl)-2,5-diphenyl tetrazolium bromide], as previously described [79]. All experiments were performed using cell cultures with a minimum viability of 90%, as determined by automated cell counting with a Countess™ 3 FL Automated Cell Counter (Invitrogen™, Thermo Fisher Scientific, Waltham, MA, USA).

Briefly, MCF7, Detroit 548, DU145, Caco2, Vero, OVCAR-3, and MOLT-4 cells were seeded into 96-well culture plates at densities of 4000, 4000, 8000, 2000, 6000, 30,000, and 80,000 cells per well, respectively, in a final volume of 200 μL of complete culture medium. Cells were incubated for 24 h at 37 °C in a humidified atmosphere containing 5% CO_2_ to allow cell attachment. Subsequently, cells were treated with different concentrations of each microalgal extract and incubated for an additional 24 h. Untreated cells were used as the negative control, whereas doxorubicin (DOX; Zytokil^®^, Laboratorios PISA, Mexico City, Mexico) and paclitaxel (PTX; Ofoxel^®^, Laboratorios PISA, Mexico City, Mexico) were included as positive controls.

Following treatment, the culture medium was carefully removed from each well and replaced with 100 μL of fresh complete culture medium. Subsequently, 10 μL of MTT solution (5 mg/mL in PBS; Bio Basic Canada Inc., Markham, ON, Canada; Cat. No. T0793) was added to each well, and the plates were incubated for 4 h at 37 °C in a humidified atmosphere containing 5% CO_2_ to allow the formation of formazan crystals. The medium was then removed, and the formazan crystals were dissolved in 100 μL of dimethyl sulfoxide (DMSO; Fisher Chemical, Fair Lawn, NJ, USA; Cat. No. D128-1). Absorbance was measured at 570 nm using a Synergy™ 2 Multi-Mode Microplate Reader (BioTek Instruments, Winooski, VT, USA).

The half-maximal inhibitory concentration (IC_50_) of each microalgal extract and the positive control drugs was determined for each cell line by nonlinear regression using a four-parameter logistic (4PL) dose–response model implemented in GraphPad Prism version 8.0.1 (GraphPad Software, San Diego, CA, USA), as previously described [80]. For each cell line and treatment, three independent biological experiments were performed, with each concentration tested in triplicate within each experiment (technical replicates). A separate four-parameter logistic dose–response curve was fitted to the data from each independent experiment, yielding three independently estimated IC_50_ values for each cell line–treatment combination. IC_50_ values are therefore reported as the mean ± SD of the three independent IC_50_ estimates. Statistical comparisons among treatments were performed using these independently estimated IC_50_ values rather than individual technical replicates. The IC_50_ values obtained for the microalgal extracts were subsequently used in all mechanistic assays.

To evaluate the time-dependent cytotoxic response, cells were treated with the corresponding IC_50_ concentration of each microalgal extract, and cell viability was determined by the MTT assay after 12, 24, 36, 48 and 60 h of exposure. Each experimental condition was evaluated in triplicate (technical replicates), and the entire experiment was independently repeated three times to ensure the reproducibility and reliability of the results.

### 4.9. Morphological Alterations Assay

Morphological alterations induced by the *Neochloris* sp. PA3 extract were evaluated by brightfield microscopy. Cells were seeded into 12-well culture plates at a density of 5 × 10^5^ cells per well in 1 mL of complete culture medium and incubated for 12 h at 37 °C in a humidified atmosphere containing 5% CO_2_ to allow cell attachment. The culture medium was then replaced with fresh complete medium containing the *Neochloris* sp. PA3 extract at the corresponding IC_50_ concentration. Untreated cells were used as the negative control, whereas DOX-treated cells at the corresponding IC_50_ concentration served as the positive control.

Representative images were acquired at 0, 4, 8, and 12 h after treatment using an Infinity 1-2 digital camera (Lumenera Corporation, Ottawa, ON, Canada) coupled to an Olympus IX71 inverted microscope (Olympus Corporation, Tokyo, Japan). Morphological evaluation focused on cell shrinkage, loss of adherence, membrane blebbing, and other morphological features associated with apoptosis.

Each experimental condition was evaluated in triplicate (technical replicates), and the entire experiment was independently repeated three times to ensure the reproducibility of the results.

### 4.10. Dual Acridine Orange/Ethidium Bromide (AO/EB) Fluorescence Staining Assay

Apoptotic and non-viable cells were evaluated using dual acridine orange/ethidium bromide (AO/EB) fluorescence staining, as previously described [81]. Cancer and non-tumorigenic cell lines were seeded into black 96-well plates (Thermo Fisher Scientific, Waltham, MA, USA; Cat. No. 167008) at a density of 4 × 10^4^ cells per well in 200 μL of complete culture medium and incubated for 12 h at 37 °C in a humidified atmosphere containing 5% CO_2_ to allow cell attachment.

The culture medium was subsequently replaced with fresh complete medium containing the *Neochloris* sp. PA3 extract, doxorubicin (DOX), or paclitaxel (PTX) at the corresponding IC_50_ concentration. Untreated cells were used as the negative control. After 4 h of treatment, cells were stained with a freshly prepared AO/EB solution containing 100 μg/mL acridine orange (AO) and 100 μg/mL ethidium bromide (EB) (Sigma-Aldrich, St. Louis, MO, USA; Cat. Nos. 318337 and E7637, respectively). A volume of 8 μL of the staining mixture was added to each well, and the cells were immediately examined by fluorescence microscopy.

Fluorescence images were acquired at 10× magnification using an EVOS™ M3000 Imaging System (Thermo Fisher Scientific, Waltham, MA, USA). AO fluorescence was visualized using the built-in Green Light Cube, whereas EB fluorescence was detected using the RFP 2.0 Light Cube. Three representative microscopic fields were examined for each experimental condition.

Cells were classified according to their nuclear morphology and fluorescence characteristics as follows: viable cells exhibited uniformly green nuclei with intact morphology; early apoptotic cells displayed yellow–green fluorescence associated with chromatin condensation; late apoptotic cells exhibited orange fluorescence accompanied by chromatin condensation and nuclear fragmentation; and non-viable cells displayed intense red fluorescence with loss of nuclear integrity. Cell classification was performed by manually scoring at least 300 cells per treatment.

Each experimental condition was evaluated in triplicate (technical replicates), and the entire experiment was independently repeated three times. The percentages of viable, early apoptotic, late apoptotic, and non-viable cells were expressed as mean ± standard deviation (SD).

### 4.11. Caspase-3/7 Activity Assay

Caspase-3/7 activity was evaluated using the CellEvent™ Caspase-3/7 Green ReadyProbes™ Reagent (Invitrogen, Carlsbad, CA, USA; Cat. No. R37111) according to the manufacturer’s instructions. Detroit 548, MCF7, Caco2, and DU145 cells were seeded into black, clear-bottom 96-well plates (Corning Inc., Corning, NY, USA; Cat. No. 3916) at the same cell densities described for the cytotoxicity assay (Section 2.6) in a final volume of 200 μL of complete culture medium. Cells were incubated for 24 h at 37 °C in a humidified atmosphere containing 5% CO_2_ to allow cell attachment.

The culture medium was subsequently replaced with fresh complete medium containing the *Neochloris* sp. PA3 extract at the corresponding IC_50_ concentration. Untreated cells were used as the negative control, whereas DOX-treated cells at the corresponding IC_50_ concentration served as the positive control. After 4 h of treatment, 2 μL of CellEvent™ Caspase-3/7 Green ReadyProbes™ Reagent was added directly to each well, and the plates were incubated for 30 min at 37 °C in a humidified atmosphere containing 5% CO_2_, protected from light.

Fluorescence images were acquired at 10× magnification using an EVOS™ M3000 Imaging System (Thermo Fisher Scientific, Bothell, WA, USA). Activated caspase-3/7 fluorescence was visualized using the Green Light Cube. Three representative microscopic fields were examined for each experimental condition.

Cells exhibiting bright green nuclear fluorescence were considered positive for caspase-3/7-associated activity. Cell classification was performed by manually scoring at least 300 cells per treatment.

Each experimental condition was evaluated in triplicate (technical replicates), and the entire experiment was independently repeated three times. Caspase-3/7 activity was expressed as the percentage of positive cells (mean ± standard deviation, SD).

### 4.12. Selectivity Index (SI) Analysis

Differential cytotoxicity between cancer and non-tumorigenic cell models was assessed by calculating the Selectivity Index (SI) for the methanolic extract of *Neochloris* sp. PA3. The SI was determined by dividing the IC_50_ value obtained for each non-tumorigenic cell model (Detroit 548 or Vero) by the corresponding IC_50_ value obtained for each cancer cell line (MCF7, Caco2, DU145, OVCAR-3, or MOLT-4). Thus, higher SI values indicate greater differential cytotoxicity toward the corresponding cancer cell line relative to the non-tumorigenic reference cell line used for the calculation.

The Detroit 548 and Vero cell lines were independently used as non-tumorigenic reference models. Because these models do not represent the diversity of normal human tissues, the SI values were interpreted only as comparative measures of cytotoxicity within the cell lines evaluated in this study [47,48].

### 4.13. Statistical Analysis

All experiments were independently performed three times, and results are presented as the mean ± standard deviation (SD), unless otherwise indicated. For cytotoxicity experiments, a separate four-parameter logistic dose–response curve was fitted for each independent biological experiment, generating three independent IC_50_ estimates for each cell line–treatment combination. Statistical comparisons of IC_50_ values were performed using these independent IC_50_ estimates as the experimental units. Differences among treatments were evaluated by one-way analysis of variance (ANOVA) followed by Tukey’s multiple comparisons test. Nonlinear regression and statistical analyses were performed using GraphPad Prism version 8.0.1 (GraphPad Software, San Diego, CA, USA). Differences were considered statistically significant at *p* < 0.05.

## 5. Conclusions

This study demonstrates that freshwater microalgae isolated from the oligotrophic environments of the Cuatro Ciénegas Basin are sources of bioactive natural products with measurable in vitro biological activity. The methanolic extracts exhibited distinct antioxidant profiles depending on the mechanism evaluated. *Neochloris* sp. PA3 showed the highest total antioxidant capacity and detectable ferrous ion-chelating activity, whereas *Chlorella* sp. PS exhibited the strongest superoxide radical scavenging activity, highlighting the complementary antioxidant properties of these microalgal isolates.

Among the four strains evaluated, *Neochloris* sp. PA3 exhibited the most favorable in vitro cytotoxic profile, with pronounced cytotoxic activity against MCF7, Caco2, and DU145 cancer cells and substantially lower cytotoxicity in the non-tumorigenic Vero and Detroit 548 cell models. The time-dependent reduction in cancer cell viability, together with apoptosis-associated morphological and nuclear alterations and increased executioner caspase-3/7 activity, supports an apoptosis-associated cellular response in PA3-treated cancer cells.

Overall, these findings identify *Neochloris* sp. PA3 as a source of natural products exhibiting antioxidant activity and differential cytotoxicity across the tested cancer and non-tumorigenic cell models. However, because the present study was conducted using a crude methanolic extract and exclusively in vitro cell-based assays, these findings should be regarded as preliminary evidence of biological activity rather than evidence of therapeutic efficacy. The bioactive constituents responsible for these effects and their underlying molecular mechanisms remain to be established. Future studies should focus on bioassay-guided fractionation and structural characterization of the active constituents, followed by mechanistic evaluation and validation in three-dimensional and appropriate in vivo models before any therapeutic relevance can be assessed.

## Figures and Tables

**Figure 1:**
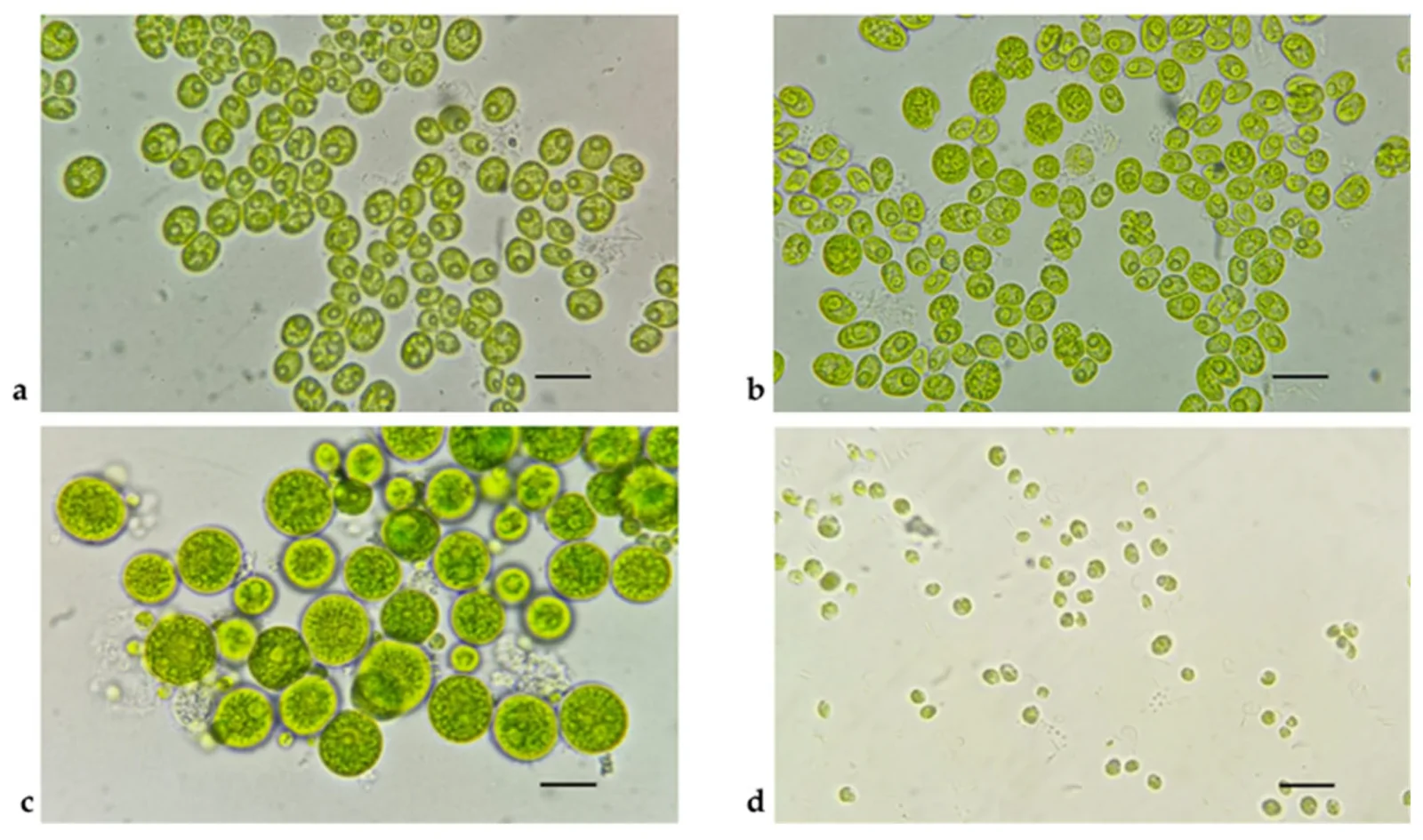
Bright-field micrographs (100× magnification) of the freshwater microalgal isolates. (**a**) *Asterarcys* sp. MOJF-1, (**b**) *Asterarcys* sp. PA2, (**c**) *Neochloris* sp. PA3, and (**d**) *Chlorella* sp. PS. Scale bars = 10 μm.

**Figure 2:**
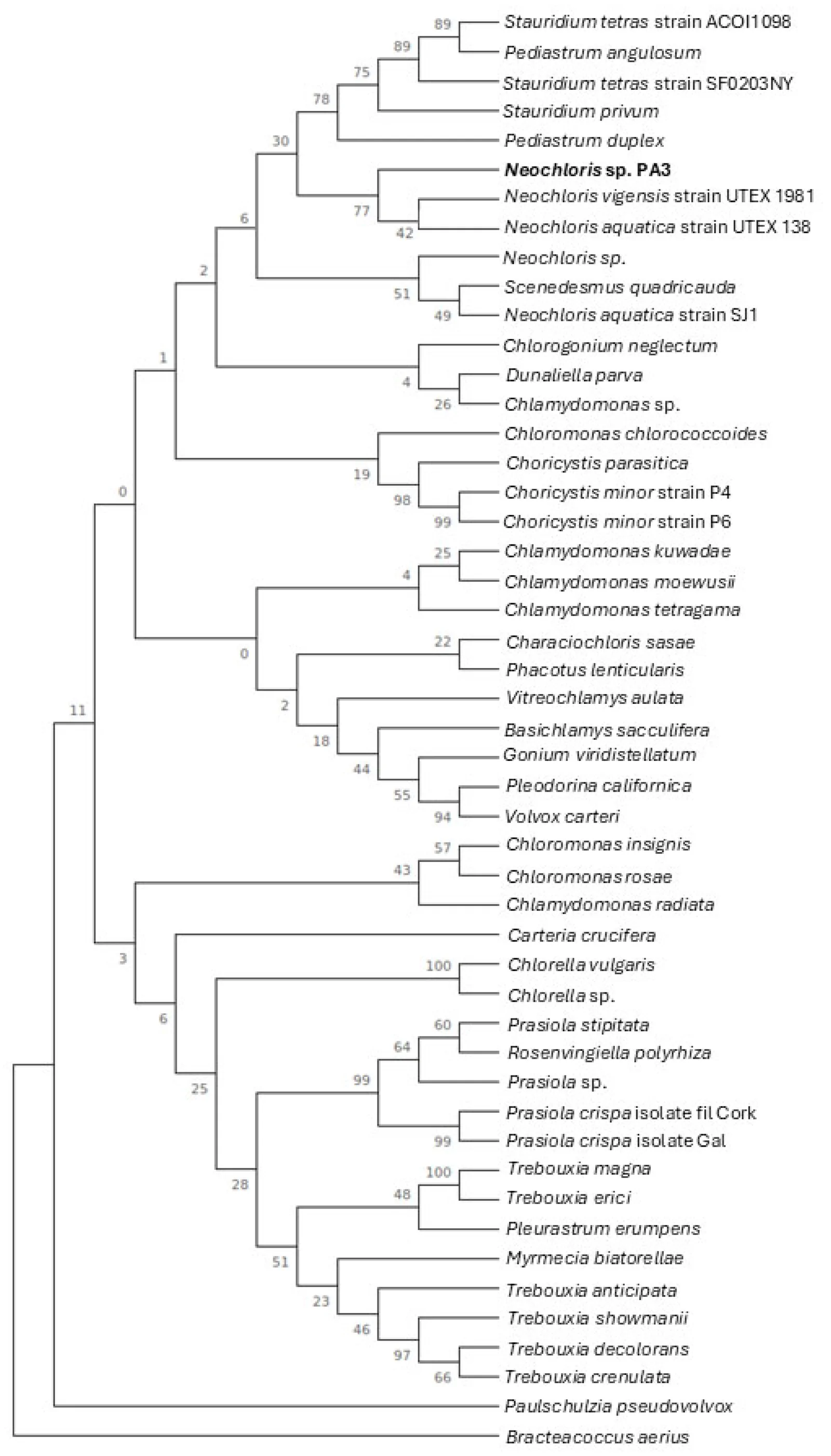
Maximum Likelihood (ML) phylogenetic tree based on partial *rbcL* gene sequences confirming the phylogenetic placement of *Neochloris* sp. PA3 among representative chlorophyte taxa. Phylogenetic reconstruction was performed using the best-fit nucleotide substitution model (GTR+G) selected in MEGA11. Bootstrap support values (%) based on 1000 replicates are indicated at the corresponding nodes. *Bracteacoccus aerius* was used as the outgroup.

**Figure 3:**
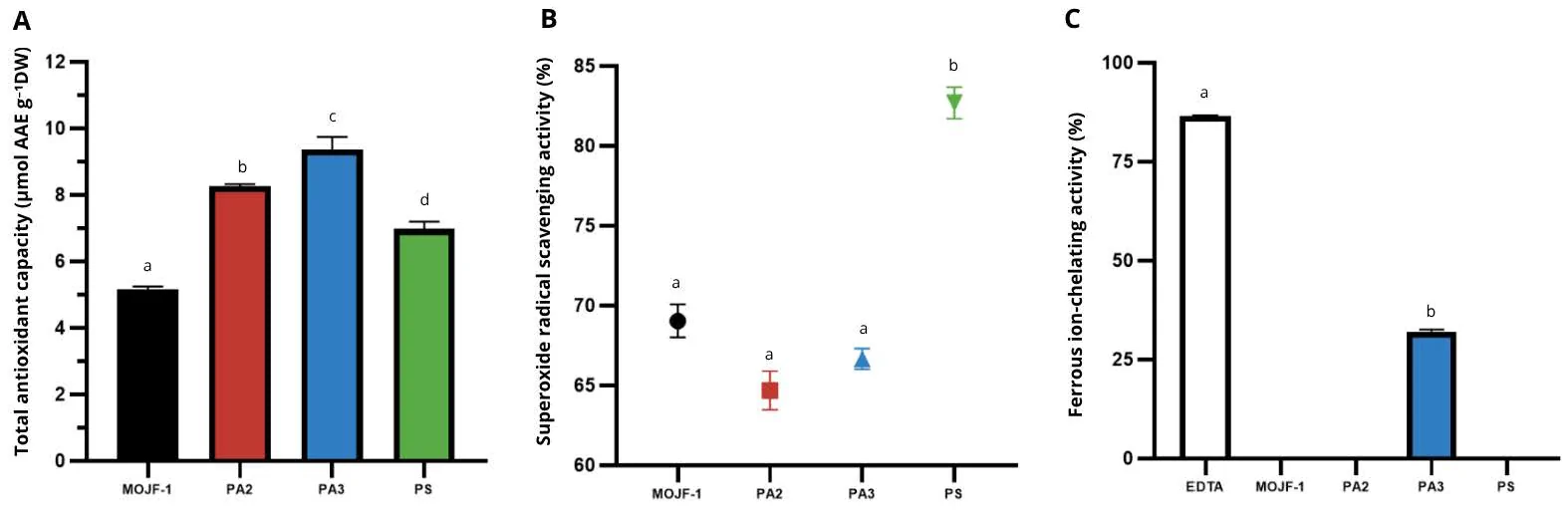
Antioxidant properties of methanolic extracts obtained from the isolated microalgae. (**A**) Total antioxidant capacity determined by the phosphomolybdenum assay and expressed as µmol ascorbic acid equivalents (AAE) g^−1^ dry weight (DW). (**B**) Superoxide radical scavenging activity of the microalgal extracts. (**C**) Ferrous ion-chelating activity of the microalgal extracts; EDTA was included as a positive control. Colors represent the different microalgal isolates: black, *Asterarcys* sp. MOJF-1; red, *Asterarcys* sp. PA2; blue, *Neochloris* sp. PA3; and green, *Chlorella* sp. PS. The white bar represents the EDTA positive control. Values represent the mean ± SD of three independent experiments. Different lowercase letters indicate statistically significant differences according to one-way ANOVA followed by Tukey’s multiple comparisons test (*p* < 0.05).

**Figure 4:**
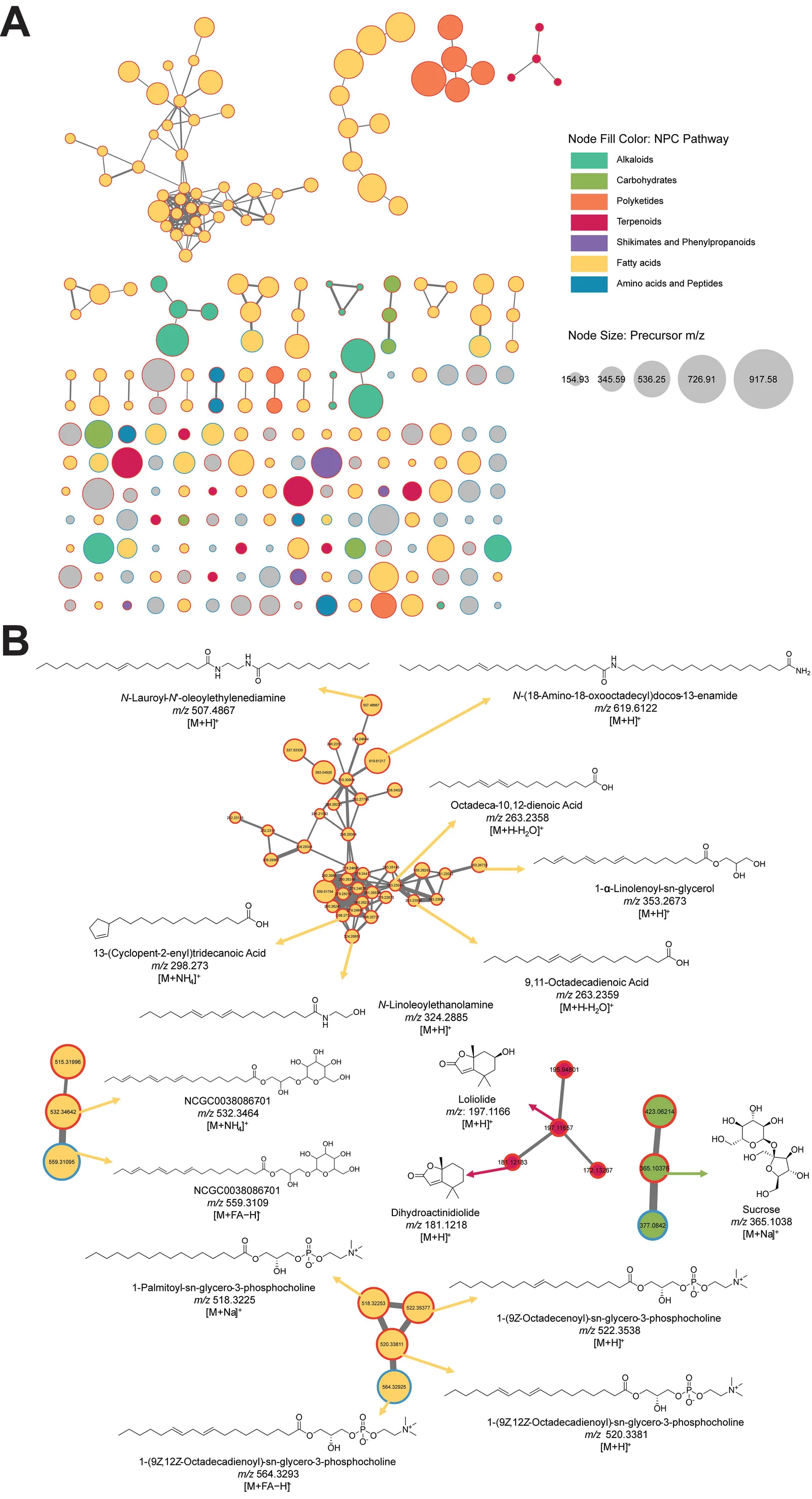
Untargeted LC-HRESI-MS/MS metabolomic profiling of the *Neochloris* sp. PA3 methanolic extract. (**A**) Molecular network generated using MS2DeepScore based on MS^2^ spectral similarity across positive and negative ionization modes. Each node represents an MS^2^-associated feature and is color-coded according to its NPClassifier pathway-level assignment. Node size is proportional to precursor *m*/*z*. (**B**) Representative molecular subnetworks highlighting selected annotated metabolites and their proposed chemical structures. Most of the annotated metabolites were lipid-related compounds (fatty acids, glycerolipids, galactolipids). Metabolite annotations were assigned by spectral-library matching and/or in silico MS/MS análisis. All annotations are included in Table S4.

**Figure 5:**
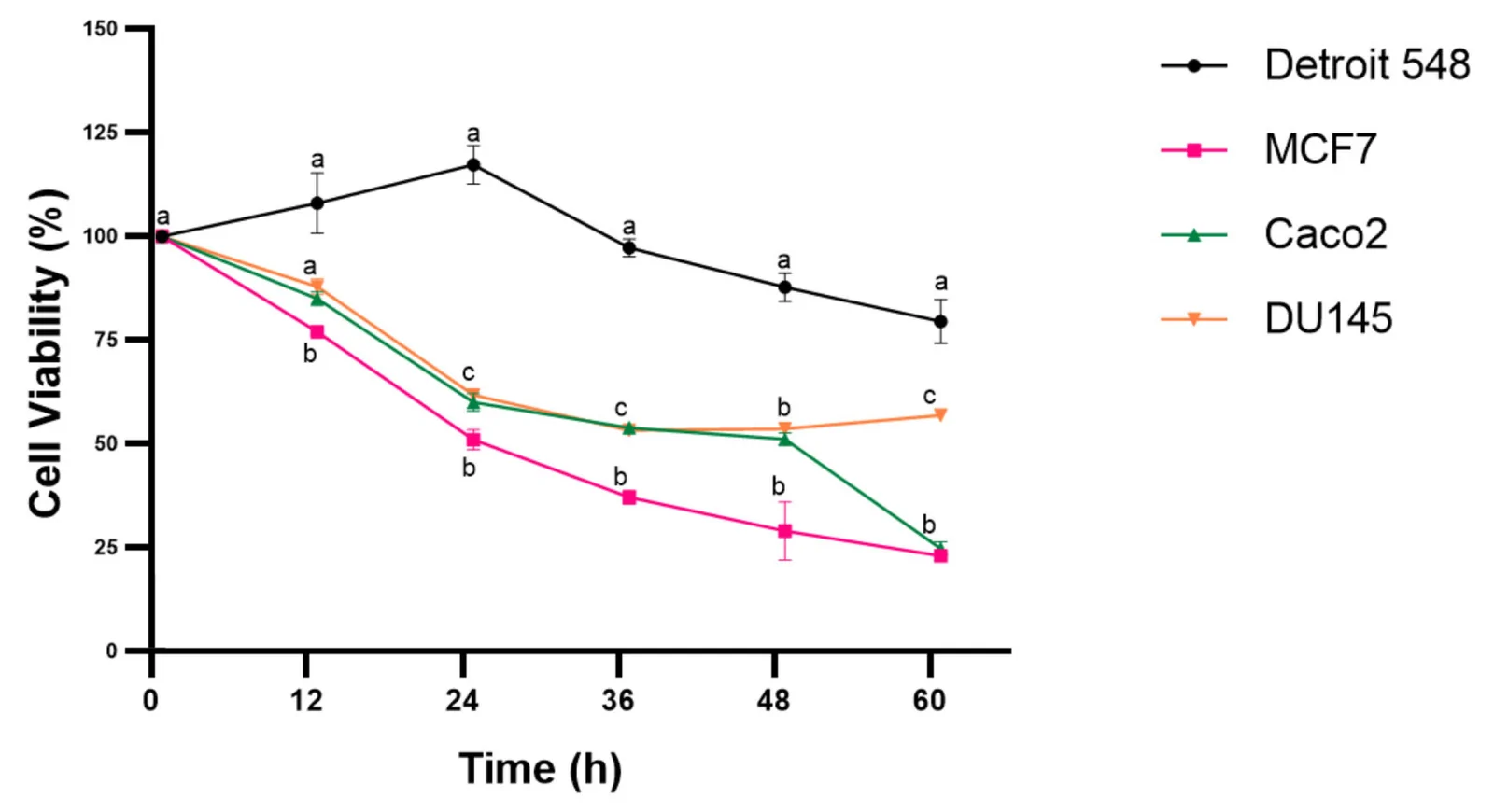
Time-dependent antiproliferative activity of the methanolic extract of *Neochloris* sp. PA3 against human cancer cell lines. MCF7, Caco2, DU145, and the non-tumorigenic Detroit 548 cell line were treated with the corresponding IC_50_ concentration of the extract, and cell viability was determined by the MTT assay after 12, 24, 36, 48, and 60 h of exposure. Cell viability is expressed as the percentage relative to untreated control cells, which were normalized to 100% at each time point. Data are presented as the mean ± standard deviation (SD) of three independent experiments performed in triplicate. Different lowercase letters at each time point indicate statistically significant differences among cell lines according to one-way ANOVA followed by Tukey’s multiple comparisons test (*p* < 0.05).

**Figure 6:**
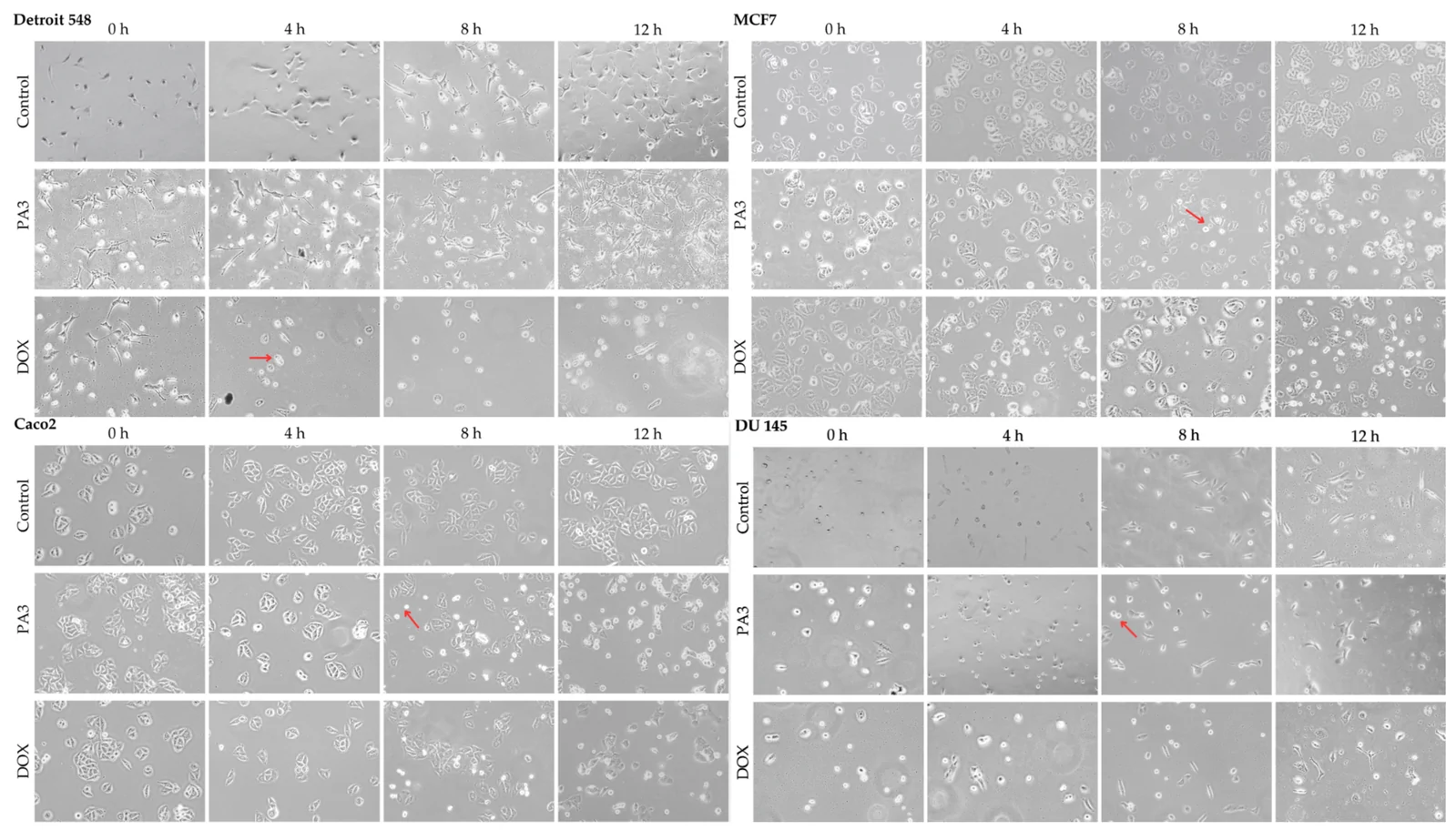
Time-dependent morphological alterations induced by the methanolic extract of *Neochloris* sp. PA3 in human cancer cell lines and non-tumorigenic Detroit 548 cells. Cells were treated with the corresponding IC_50_ concentration of the extract (PA3) or doxorubicin (DOX, positive control), whereas untreated cells served as the negative control. Representative bright-field micrographs were acquired at 0, 4, 8, and 12 h after treatment. Red arrows indicate representative rounded cells displaying loss of adherence and increased refringence, morphological features commonly associated with apoptosis.

**Figure 7:**
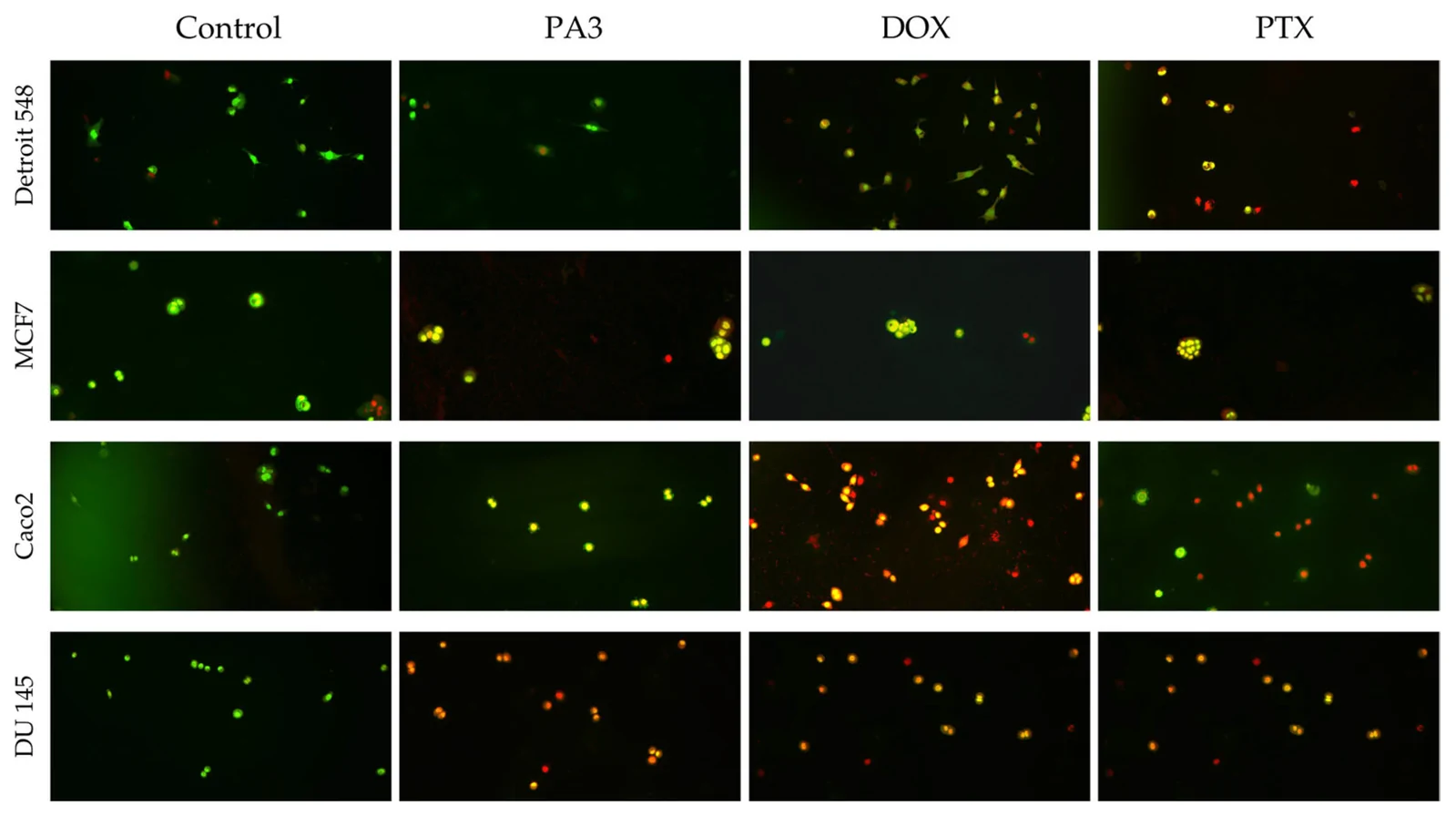
Representative fluorescence micrographs of dual acridine orange/ethidium bromide (AO/EB) staining in Detroit 548, MCF7, Caco2, and DU145 cells following 4 h of treatment with the methanolic extract of *Neochloris* sp. PA3, doxorubicin (DOX), or paclitaxel (PTX). Untreated cells exhibited predominantly viable cells with uniformly green nuclei. PA3-treated cancer cells displayed nuclear alterations, including chromatin condensation, nuclear fragmentation, and yellow-green to orange fluorescence, features consistent with early and late apoptotic cell death.

**Figure 8:**
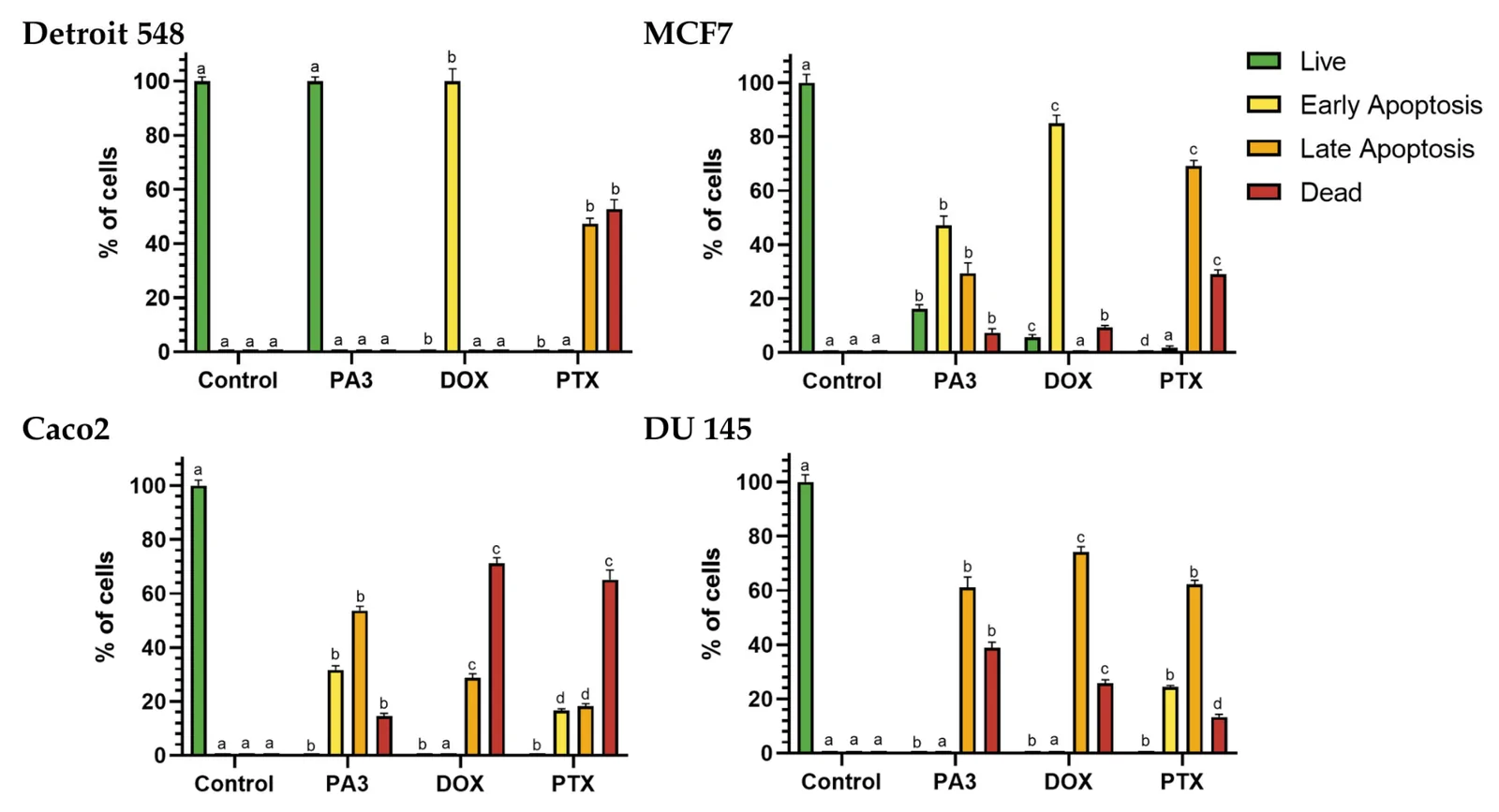
Quantitative analysis of cell viability and nuclear morphology following dual acridine orange/ethidium bromide (AO/EB) staining. Cells were classified as viable (green), early apoptotic-like (yellow-green), late apoptotic-like (orange), or non-viable (red) based on their fluorescence pattern and nuclear morphology after 4 h of treatment with the methanolic extract of *Neochloris* sp. PA3, doxorubicin (DOX), or paclitaxel (PTX). Data represent the percentage of cells manually scored from at least 300 cells per treatment and are expressed as the mean ± SD of three independent experiments. Different lowercase letters indicate statistically significant differences among treatments within each cellular category according to one-way ANOVA followed by Tukey’s multiple comparisons test (*p* < 0.05).

**Figure 9:**
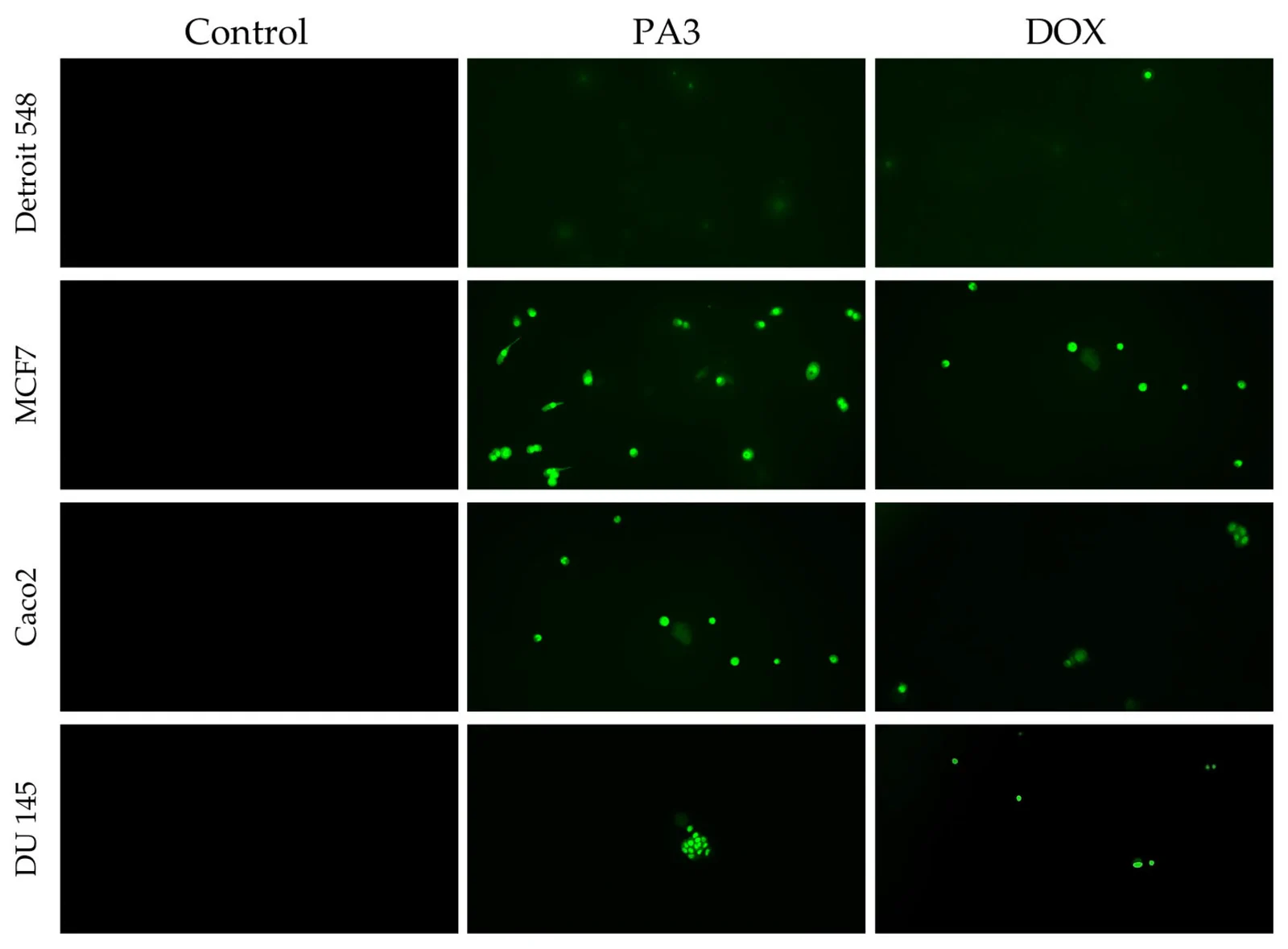
Representative fluorescence micrographs showing caspase-3/7 activity following treatment with the methanolic extract of *Neochloris* sp. PA3 or doxorubicin (DOX). MCF7, Caco2, DU145, and Detroit 548 cells were incubated with the corresponding IC_50_ concentration of each treatment for 4 h and stained with the CellEvent™ Caspase-3/7 Green Detection Reagent. Green fluorescence indicates cells positive for caspase-3/7 associated activity. Untreated cells served as the negative control.

**Figure 10:**
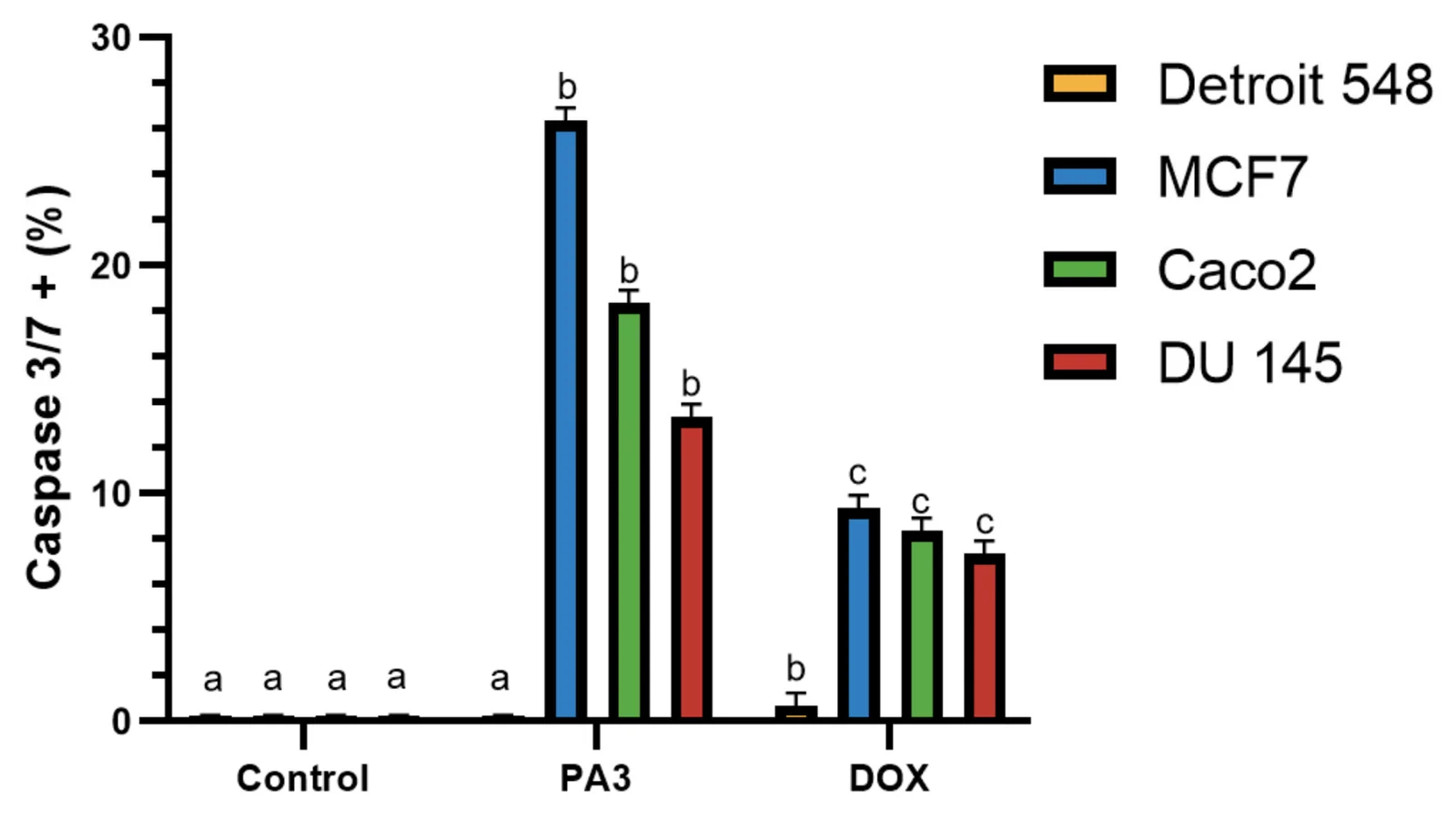
Quantification of caspase-3/7-positive cells following treatment with the methanolic extract of *Neochloris* sp. PA3 or doxorubicin (DOX). Data represent the percentage of caspase-3/7-positive cells after 4 h of treatment and are expressed as the mean ± SD of three independent experiments. Different lowercase letters indicate statistically significant differences among treatments within each cell line according to one-way ANOVA followed by Tukey’s multiple comparisons test (*p* < 0.05).

**Table 1:** Half-maximal inhibitory concentration (IC_50_) values of methanolic extracts obtained from four freshwater microalgal isolates against human cancer and non-tumorigenic cell lines. Cells were exposed to each extract for 24 h, and cell viability was determined using the MTT assay. Doxorubicin (DOX) and paclitaxel (PTX) were included as reference anticancer drugs. Data are presented as the mean ± standard deviation (SD) of IC_50_ values independently estimated from three biological experiments, with each concentration tested in triplicate within each experiment. Different superscript letters within the same column indicate statistically significant differences among the independently estimated IC_50_ values according to one-way ANOVA followed by Tukey’s multiple comparisons test (*p* < 0.05).

Cancer Cell Lines	PA3	PA2	PS	MOJF-1	DOX	PTX
MCF7	17.1 ± 1.6 ^a^	43.0 ± 5.2 ^b,c^	43.4 ± 5.7 ^a,b^	64.7 ± 7.0 ^a^	0.67 ± 0.1 ^a^	11.3 ± 3.9 ^c^
Caco2	19.0 ± 0.9 ^a^	22.1 ± 2.8 ^a,b^	26.9 ± 3.3 ^a^	18.3 ± 1.0 ^a^	6.5 ± 0.2 ^b^	0.2 ± 0.08 ^a^
DU 145	19.1 ± 1.2 ^a^	14.5 ± 1.5 ^a^	68.1 ± 5.3 ^a,b^	23.5 ± 2.6 ^a^	102.6 ± 9.3 ^d^	1491 ± 79 ^e^
MOLT-4	27.2 ±3.2 ^a^	29.9 ± 3.3 ^a,b^	86.7 ± 7.1 ^a,b^	44.0 ± 3.3 ^a^	34.8 ± 2.2 ^c^	94.1 ± 17.3 ^d^
OVCAR-3	200.2 ± 27.9 ^b^	59.9 ± 2.7 ^c^	825.7 ± 147.6 ^c^	410.2 ± 67.6 ^b^	1902.4 ± 547.8 ^e^	4632 ± 1086 ^f^
Non-tumorigenic cell lines						
Vero	170.9 ± 11.5 ^b^	153.3 ± 16.7 ^d^	64.1 ± 3.7 ^a,b^	179.5 ± 11.3 ^c^	1.98 ± 0.6 ^a^	5.19 ± 1.1 ^b^
Detroit 548	192.1 ± 13.9 ^b^	143.3 ± 9.5 ^d^	189.7 ± 16.5 ^b^	189.9 ± 21.2 ^c^	1.21 ± 0.2 ^a^	0.66 ± 0.2 ^a^

**Table 2:** Cytotoxicity and selectivity indices of the *Neochloris* sp. PA3 methanolic extract in cancer and non-tumorigenic cell lines. SI values were calculated by dividing the IC_50_ obtained in each non-tumorigenic cell model (Vero or Detroit 548) by the corresponding IC_50_ obtained for each cancer cell line. Higher SI values indicate greater differential cytotoxicity toward the corresponding cancer cell line relative to the tested non-tumorigenic cell line. -, not applicable.

Cancer and Non-Tumorigenic Cell Lines	IC_50_ (μg/mL)	SI (Vero)	SI (Detroit 548)
MCF7	17.1 ± 1.6	9.99	11.23
Caco2	19.0 ± 0.9	8.99	10.11
DU 145	19.1 ± 1.2	8.95	10.06
MOLT-4	27.2 ± 3.2	6.28	7.06
OVCAR-3	200.2 ± 27.9	0.85	0.96
Vero	170.9 ± 11.5	-	-
Detroit 548	192.1 ± 13.9	-	-

## Data Availability

The data supporting the findings of this study are available within the article and its Supplementary Materials. The partial *rbcL* nucleotide sequences generated in this study have been deposited in the NCBI GenBank database under accession numbers PZ759594 (*Neochloris* sp. PA3), PZ759593 (*Asterarcys* sp. MOJF-1), PZ776196 (*Asterarcys* sp. PA2), and PZ759595 (*Chlorella* sp. PS). Annotated metabolites in *Neochloris* sp. PA3 GNPS2. The raw LC–MS2 datasets are stored in the GNPS/MassIVE public repository with accession number MSV000103369. Details about the feature-based molecular networking parameters and spectral matching for both positive and negative modes can be accessed at these links: https://gnps2.org/status?task=2ca077b1d8c44b008e394a24e79b8ee2 (accessed on 10 October 2026) and https://gnps2.org/status?task=4157340f5aac4a208a0c051f63803d00 (accessed on 10 October 2026). Additional experimental data are available from the corresponding author upon reasonable request.

## Supplementary Materials

The following supporting information can be downloaded at https://www.mdpi.com/article/10.3390/ijms27209258/s1.
