# Supplementary material for: Neochloris sp. PA3, an Oligotrophic Desert Oasis Microalga, Exhibits Differential Cytotoxicity and Apoptosis-Associated Effects in Human Cancer Cells
Source: Int J Mol Sci. 2026 Oct 18;27(20):9258. doi: 10.3390/ijms27209258 (PMC13705518; doi:10.3390/ijms27209258)
Supplement: Supplementary file 1 [file ijms-27-09258-s001.zip › Supplementary Figures.pdf]

Supplementary Material

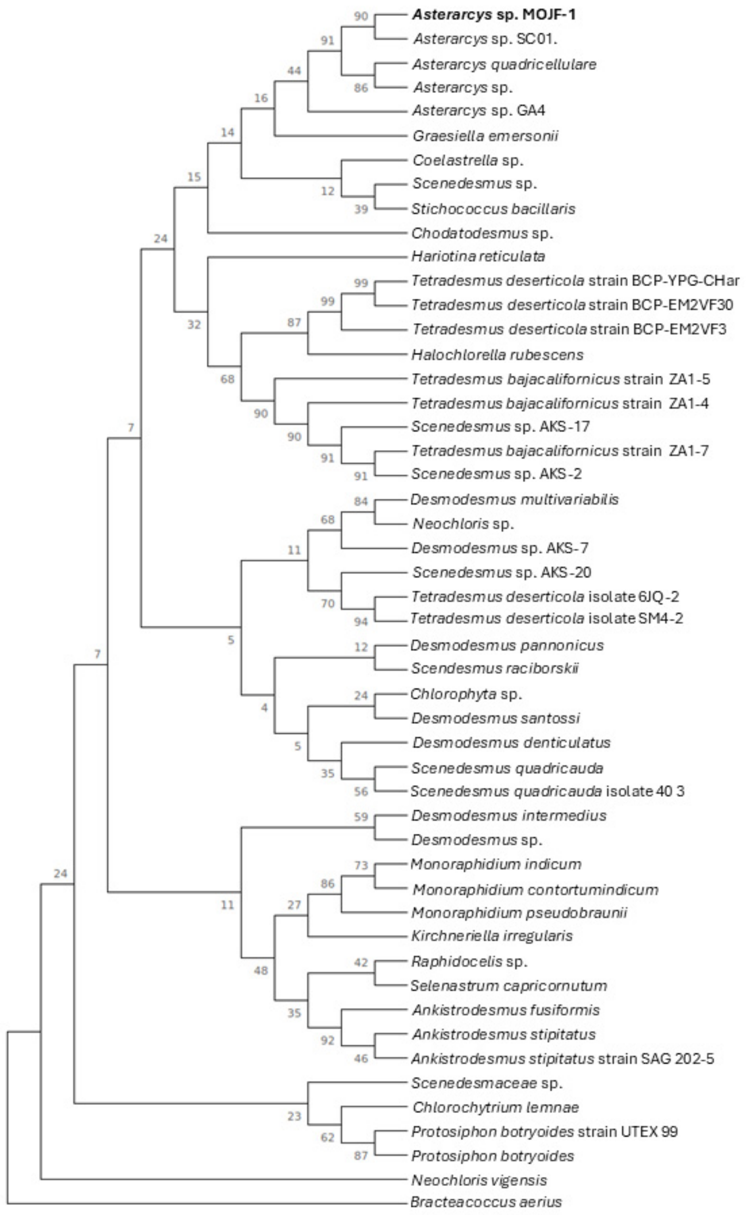

Figure S1. Maximum Likelihood (ML) phylogenetic tree based on partial *rbcL* gene sequences showing the phylogenetic placement of *Asterarcys* sp. MOJF-1 among representative chlorophyte taxa. Phylogenetic reconstruction was performed using the General Time Reversible model with a discrete Gamma distribution (GTR+G). Bootstrap support values (%) based on 1000 replicates are indicated at the corresponding nodes. *Bracteacoccus aerius* was used as the outgroup.

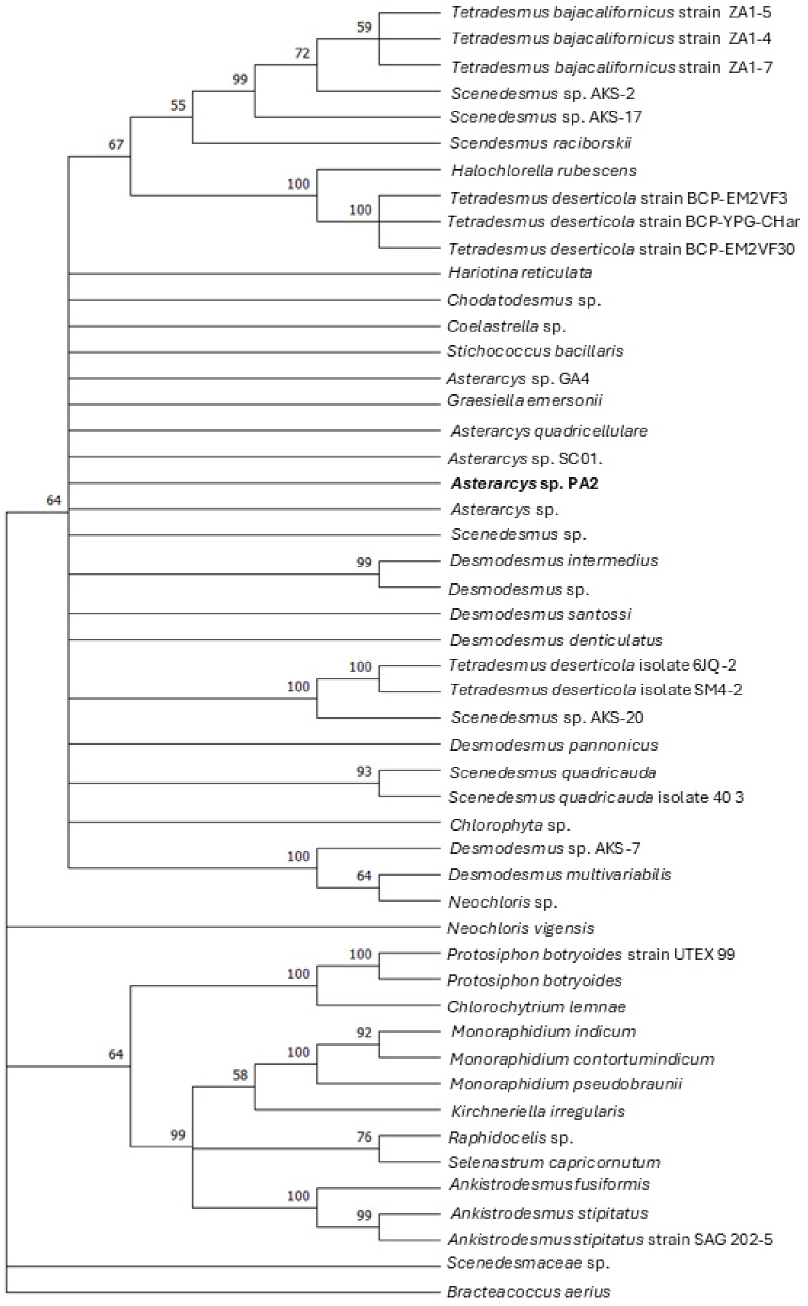

Figure S2. Maximum Likelihood (ML) phylogenetic tree based on partial *rbcL* gene sequences showing the phylogenetic placement of *Asterarcys* sp. PA2 among representative chlorophyte taxa. Phylogenetic reconstruction was performed using the General Time Reversible model with a discrete Gamma distribution (GTR+G). Bootstrap support values (%) based on 1000 replicates are indicated at the corresponding nodes. *Bracteacoccus aerius* was used as the outgroup.

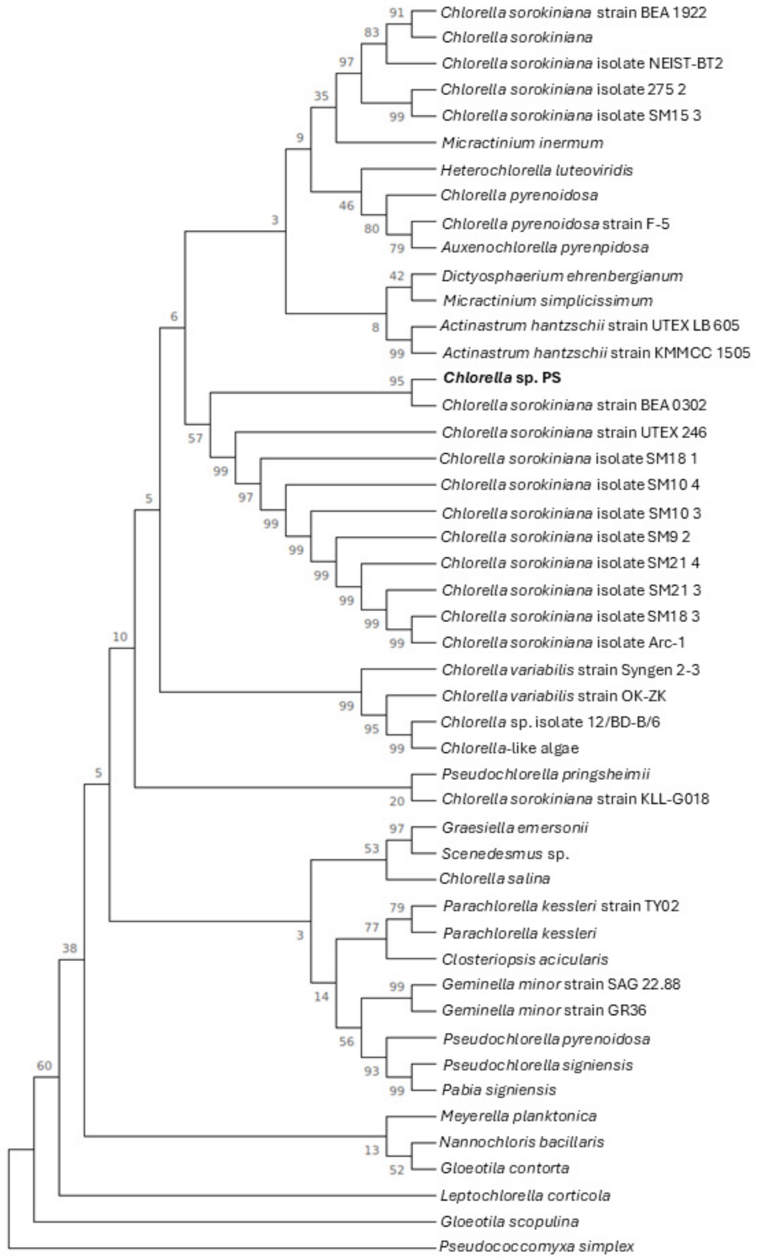

Figure S3. Maximum Likelihood (ML) phylogenetic tree based on partial *rbcL* gene sequences showing the phylogenetic placement of *Chlorella* sp. PS among representative chlorophyte taxa. Phylogenetic reconstruction was performed using the General Time Reversible model with a discrete Gamma distribution (GTR+G). Bootstrap support values (%) based on 1000 replicates are indicated at the corresponding nodes. *Pseudococcomyxa simplex* was used as the outgroup.

Table S1. Reference *rbcL* sequences retrieved from NCBI GenBank and used for phylogenetic analyses of *Asterarcys* sp. MOJF-1 and *Asterarcys* sp. PA2.

	Taxon	NCBI GenBank	Isolation source	Reference
1	*Ankistrodesmus fusiformis*	KT833576.1	Järnsjön, Lake in Sweden	[1]
2	*Ankistrodesmus stipitatus*	KT355749.1	Minas Gerais, Brazil	[2]
3	*Ankistrodesmus stipitatus* strain SAG 202-5	EF113406.1		Data deposited in NCBI
4	*Asterarcys quadricellulare*	OK626438.1	Water pond, Greece	
5	*Asterarcys* sp. GA4	MW363984.1	Vaal river, South Africa	
6	*Asterarcys* sp. NEIST BT-13	MH324932.1	Fresh water, India	
7	*Asterarcys* sp. SC01	MW560279.1	Fresh water, India	
8	*Bracteacoccus aerius*	JQ259861.1		
9	*Chlorochytrium lemnae*	HE860265.1		[3]
10	*Chlorophyta* sp.	MF278351.1	Coal mine drainage manganese removal bed Mn oxide layer, USA.	Data deposited in NCBI
11	*Chodatodesmus* sp.	OL770103.1	Lake Baikal, Russia	
12	*Coelastrella* sp.	MK257133.1	Gray forest soil, Russia	
13	*Desmodesmus denticulatus*	GU192429.1	Tower pond, USA	[4]
14	*Desmodesmus intermedius*	MK842152.1		Data deposited in NCBI
15	*Desmodesmus multivariabilis*	GU192431.1	Tower pond, USA	[4]
16	*Desmodesmus pannonicus*	MK257140.1	Meadow soil, Russia	Data deposited in NCBI
17	*Desmodesmus santosii*	GU192418.1	Tower pond, USA	
18	*Desmodesmus* sp. AKS-7	KF975595.1		
19	*Desmodesmus* sp. Tow8/18P-4W	GU192430.1	Tower pond, USA	[4]
20	*Graesiella emersonii*	OP605746.1	South Korea	[5]
21	*Halochlorella rubescens*	MT655944.1		[6]
22	*Hariotina reticulata*	KY792652.1		Data deposited in NCBI
23	*Kirchneriella irregularis*	KT355752.1	Brazil	[1]
24	*Monoraphidium contortum*	KT833562.1		
25	*Monoraphidium indicum*	KT833568.1		
26	*Monoraphidium pseudobraunii*	KT355751.1		
27	*Neochloris* sp.	EF012704.1		Data deposited in NCBI
28	*Neochloris vigensis*	EF113457.1		
29	*Protosiphon botryoides* isolate FRT2000	JN880462.1		
30	*Protosiphon botryoides* strain UTEX 99	EF113465.1		
31	*Raphidocelis* sp.	LC472548.1		
32	*Scenedesmaceae* sp.	HG514362.1	Lake, Antarctica	[7]
33	*Scenedesmus quadricauda*	AB084332.1		[8]
34	*Scenedesmus quadricauda* isolate 40_3	KT777979.1		[9]
35	*Scenedesmus raciborskii*	HG514380.1		[7]
36	*Scenedesmus* sp. AKS-17	KF975602.1		Data deposited in NCBI
37	*Scenedesmus* sp. AKS-2	KF975592.1		
38	*Scenedesmus* sp. AKS-20	KF975604.1		
39	*Scenedesmus* sp. 'almeriensis'	MG257492.1	Spain	[10]
40	*Selenastrum capricornutum*	LC200423.1		

41	*Stichococcus bacillaris*	EF113475.1		Data deposited in NCBI
42	*Tetradesmus bajacalifornicus* strain ZA1-4	HQ246355.1		[11]
43	*Tetradesmus bajacalifornicus* strain ZA1-5	HQ246356.1		
44	*Tetradesmus bajacalifornicus* strain ZA1-7	HQ246357.1		
45	*Tetradesmus deserticola* isolate 6JQ-2	KT777976.1		[9]
46	*Tetradesmus deserticola* isolate SM4-2	KT777973.1		
47	*Tetradesmus deserticola* strain BCP-EM2VF3	HQ246359.1		[11]
48	*Tetradesmus deserticola* strain BCP-EM2VF30	HQ246358.1		
49	*Tetradesmus deserticola* strain BCP-YPG-Char	HQ246361.1		

Table S2. Reference *rbcL* sequences retrieved from NCBI GenBank and used for phylogenetic analysis of *Neochloris* sp. PA3.

	**Taxon**	**NCBI GenBank**	**Isolation source**	**Reference**
1	*Basichlamys sacculifera*	D63430.1		[12]
2	*Bracteacoccus aerius*	JQ259861.1		Data deposited in NCBI
3	*Chlamydomonas moewusii*	M15842.1		[13]
4	*Carteria crucifera*	D63431.1		[12]
5	*Characiochloris sasae*	AB084338.1		[8]
6	*Chlamydomonas kuwadae*	AB084334.1		
7	*Chlamydomonas radiata*	AJ001878.1		[14]
8	*Chlamydomonas* sp. AKS-10	KF975597.1		Data deposited in NCBI
9	*Chlamydomonas tetragama*	AJ001880.1		[14]
10	*Chlorella* sp.	EF012700.1		Data deposited in NCBI
11	*Chlorella vulgaris*	AF499684.1	Freshwater, Netherlands	[15]
12	*Chlorogonium neglectum*	AB010243.1		[16]
13	*Chloromonas chlorococcoides*	AB101508.1	Soil, America	[17]
14	*Chloromonas insignis*	AB022226.1		[18]
15	*Chloromonas rosae*	AB022532.2	Freshwater, Norway	
16	*Choricystis* minor strain P4	EF012702.1		Data deposited in NCBI
17	*Choricystis* minor strain P6	EF012703.1		
18	*Choricystis parasitica*	DQ219819.1	Freshwater, Germany	
19	*Dunaliella parva*	AJ001877.1		[14]
20	*Gonium viridistellatum*	D86831.1		[19]
21	*Myrmecia biatorellae*	AF499685.1	Phycobiont, Austria	[15]
22	*Neochloris aquatica* strain SJ1	KU877408.1	India	Data deposited in NCBI
23	*Neochloris aquatica* strain UTEX 138	EF113456.1		
24	*Neochloris* sp.	EF012704.1		
25	*Neochloris vigensis*	EF113457.1		
26	*Paulschulzia pseudovolvox*	D86837.1		[19]

27	*Pediastrum angulosum*	EF078324.1		Data deposited in NCBI
28	*Pediastrum duplex*	AB084333.1		[8]
29	*Phacotus lenticularis*	AJ001883.1		[14]
30	*Pleodorina californica*	D63439.1		[19]
31	*Pleurastrum erumpens*	AF189068.1		[20]
32	*Prasiola crispa* isolate fil_Cork	AY694196.1		[21]
33	*Prasiola crispa* isolate Gal	AY694195.1		
34	*Prasiola* sp.	AY694197.1		
35	*Prasiola stipitata*	AY694192.1		
36	*Rosenvingiella polyrhiza*	AY694206.1		
37	*Scenedesmus quadricauda*	AB084332.1		[8]
38	*Stauridium privum*	EF078392.1		Data deposited in NCBI
39	*Stauridium tetras* strain ACOI1098	EF078378.1		
40	*Stauridium tetras* strain SF0203NY	EF078330.1		
41	*Trebouxia anticipata*	AF189069.1		[20]
42	*Trebouxia crenulata*	AJ969639.2		Data deposited in NCBI
43	*Trebouxia decolorans*	AM159504.1	Switzerland	
44	*Trebouxia erici*	AJ969631.1		
45	*Trebouxia magna*	AJ969630.1		
46	*Trebouxia showmanii*	AJ969661.1		
47	*Vitreochlamys aulata*	AB050487.2	Japan: Kanagawa, Atsugi	[22]
48	*Volvox carteri*	D63446.1		[12]

Table S3. Reference *rbcL* sequences retrieved from NCBI GenBank and used for phylogenetic analysis of *Chlorella* sp. PS.

	**Taxon**	**NCBI GenBank**	**Isolation source**	**Reference**
1	*Actinastrum hantzschii* strain KMMCC 1505	JQ315472.1		Data deposited in NCBI
2	*Actinastrum hantzschii* strain UTEX LB 605	EF113405.1		
3	*Auxenochlorella pyrenoidosa*	ON873881.1		
4	*Chlorella pyrenoidosa* isolate 8_3	KM514877.1		[23]
5	*Chlorella pyrenoidosa* strain F-5	EU038282.1		Data deposited in NCBI
6	*Chlorella salina*	MN519695.1	Sea water, South Korea	[24]
7	*Chlorella sorokiniana*	MK842150.1		Data deposited in NCBI
8	*Chlorella sorokiniana* isolate 275_2	KT777994.1		[9]
9	*Chlorella sorokiniana* isolate Arc-1	ON602073.1	China	Data deposited in NCBI
10	*Chlorella sorokiniana* isolate NEIST-BT2	MF600427.1	Fresh water, India	
11	*Chlorella sorokiniana* isolate SM10_3	KM514905.1		[23]
12	*Chlorella sorokiniana* isolate SM10_4	KM514906.1		
13	*Chlorella sorokiniana* isolate SM15_3	KM514908.1		
14	*Chlorella sorokiniana* isolate SM18_1	KM514916.1		
15	*Chlorella sorokiniana* isolate SM18_3	KM514917.1		
16	*Chlorella sorokiniana* isolate SM21_3	KM514902.1		

17	*Chlorella sorokiniana* isolate SM21_4	KM514903.1		
18	*Chlorella sorokiniana* isolate SM9_2	KM514904.1		
19	*Chlorella sorokiniana* strain BEA 0302	OM991974.1	Freshwater, Spain	Data deposited in NCBI
20	*Chlorella sorokiniana* strain BEA 1922	OM991975.1	Water sample, Spain	
21	*Chlorella sorokiniana* strain KLL-G018	KP698022.1	Lake, Israel	[25]
22	*Chlorella sorokiniana* strain UTEX 246	EF113429.1		Data deposited in NCBI
23	*Chlorella* sp.	KJ446805.1	Switzerland	[26]
24	*Chlorella variabilis*	AB260904.1	Japan	Data deposited in NCBI
25	*Chlorella variabilis* strain OK1-ZK	AB260899.1	Japan	
26	*Chlorella variabilis* isolate 32-3	MT680610.1	China	
27	*Chlorella*-like algae	M74441.1		[27]
28	*Closteriopsis acicularis*	EF113433.1		Data deposited in NCBI
29	*Dictyosphaerium ehrenbergianum*	KM514864.1		[23]
30	*Geminella minor* strain GR36	EF113440.1		Data deposited in NCBI
31	*Geminella minor* strain SAG 22.88	EF113439.1		
32	*Gloeotila contorta*	EF113444.1		
33	*Gloeotila scopulina*	HQ613263.1	Fresh water, United Kingdom	[28]
34	*Graesiella emersonii*	OP605746.1	South Korea	[5]
35	*Heterochlorella luteoviridis*	KM514870.1		[23]
36	*Leptochlorella corticola*	HE984583.1	Slovenia	[29]
37	*Meyerella planktonica*	AY543048.1	Lake, USA	[30]
38	*Micractinium inermum*	MT550637.1	Freshwater system, Ecuador	Data deposited in NCBI
39	*Micractinium simplicissimum*	MW880710.1	Water, Russia	[31]
40	*Nannochloris bacillaris*	AB383150.1		Data deposited in NCBI
41	*Parachlorella kessleri*	AB260912.1		
42	*Parachlorella kessleri* strain TY02	KX021358.1	Soil, China	
43	*Pseudochlorella pringsheimii*	MK295220.1	Fresh water, China	
44	*Pseudochlorella pyrenoidosa*	MW077585.1		[32]
45	*Pseudochlorella signiensis*	MW077584.1		
46	*Pseudococcomyxa simplex*	EF589155.1		Data deposited in NCBI
47	*Scenedesmus* sp.	KF975604.1		

1. Garcia da Silva, T., Bock, C., Sant'Anna, C. L., Bagatini, I. L., Wodniok, S., Vieira, A. A. H. Selenastraceae (Sphaeropleales, Chlorophyceae): rbcL, 18S rDNA and ITS-2 secondary structure enlightens traditional taxonomy, with description of two new genera, Messastrum gen. nov. and Curvastrum gen. nov. *Fottea* **2017**, *17*, 1-19.
2. Garcia, T.S., Bock, C., Bagatini, I. L., Archanjo, N. C. P., Sant'Anna, C. L., Vieira, A. A. H. Hidden diversity in Selenastraceae (Sphaeropleales, Chlorophyceae): Molecular phylogeny revealed a nannoplanktonic species, with the description of *Viridiparva madeirensis* gen. et sp. nov. *Algal research* **2022**, *68*, 102886.
3. Škaloud, P., Kalina, T., Nemjová, K., De Clerck, O., Leliaert, F. Morphology and phylogenetic position of the freshwater green microalgae *Chlorochytrium* (Chlorophyceae) and *Scotinosphaera* (Scotinosphaerales, ord. nov., Ulvophyceae). *Journal of Phycology* **2013**, *49*, 115-129.
4. Fawley, M.W., Fawley, K. P., Hegewald, E. Taxonomy of *Desmodesmus serratus* (Chlorophyceae, Chlorophyta) and related taxa on the basis of morphological and DNA sequence data. *Phycologia* **2011**, *50*, 23-56.

5. Kang, N.S., Cho, K., An, S. M., Kim, E. S., Ki, H., Lee, C. H., Hong, J. W. Taxonomic and biochemical characterization of microalga *Graesiella emersonii* GEGS21 for its potential to become feedstock for biofuels and bioproducts. *Energies* **2022**, *15*, 8725.
6. Jo, S.W., Hong, J. W., Do, J. M., Na, H., Kim, J. J., Park, S. I., Yoon, H. S. Nitrogen deficiency-dependent abiotic stress enhances carotenoid production in indigenous green microalga *Scenedesmus rubescens* KNUA042, for use as a potential resource of high value products. *Sustainability* **2020**, *12*, 5445.
7. Sciuto, K., Lewis, L. A., Verleyen, E., Moro, I., La Rocca, N. *Chodatodesmus australis* sp. nov.(Scenedesmaceae, Chlorophyta) from Antarctica, with the emended description of the genus *Chodatodesmus*, and circumscription of *Flechtneria rotunda* gen. et sp. nov. *Journal of Phycology* **2015**, *51*, 1172-1188.
8. Nozaki, H., Onishi, K., Morita, E. Differences in pyrenoid morphology are correlated with differences in the rbcL genes of members of the *Chloromonas* lineage (Volvocales, Chlorophyceae). *Journal of Molecular Evolution* **2002**, *55*, 414-430.
9. Zou, S., Fei, C., Wang, C., Gao, Z., Bao, Y., He, M., Wang, C. How DNA barcoding can be more effective in microalgae identification: a case of cryptic diversity revelation in *Scenedesmus* (Chlorophyceae). *Scientific reports* **2016**, *6*, 36822.
10. Beatrice-Lindner, P., Garrido-Cardenas, J. A., Sepulveda, C., Acien-Fernandez, F. G. A new approach for detection and quantification of microalgae in industrial-scale microalgal cultures. *Applied microbiology and biotechnology* **2018**, *102*, 8429-8436.
11. Hall, J.D., Fucikova, K., Lo, C., Lewis, L. A. Karol, K. G. An assessment of proposed DNA barcodes in freshwater green algae. *Cryptogram* **2010**, *31*, 529-555.
12. Nozaki, H., Itoh, M., Sano, R., Uchida, H., Watanabe, M. M., Kuroiwa, T. Phylogenetic relationships within the colonial Volvocales (Chlorophyta) inferred from *rbcL* gene sequence data. *Journal of Phycology* **1995**, *31*, 970-979.
13. Yang, R.C., Dove, M., Seligy, V. L., Lemieux, C., Tunnel, M., Narang, S. A. Complete nucleotide sequence and mRNA-mapping of the large subunit gene of ribulose-1, 5-bisphosphate carboxylase/oxygenase (Rubisco) from *Chlamydomonas moewusii*. *Gene* **1986**, *50*, 259-270.
14. Hepperle, D., Nozaki, H., Hohenberger, S., Huss, V. A. R., Morita, E., Krienitz, L. Phylogenetic position of the *Phacotaceae* within the Chlamydophyceae as revealed by analysis of 18S rDNA and *rbcL* sequences. *Journal of Molecular Evolution* **1998**, *47*, 420-430.
15. Hayden, H.S., & Waaland, J. R. Phylogenetic systematics of the ulvaceae (ulvales, ulvophyceae) using chloroplast and nuclear dna sequences 1. *Journal of Phycology* **2002**, *38*, 1200-1212.
16. Nozaki, H., Ohta, N., Morita, E., Watanabe, M. M. Toward a natural system of species in *Chlorogonium* (Volvocales, Chlorophyta): a combined analysis of morphological and rbcL gene sequence data. *Journal of Phycology* **1998**, *34*, 1024-1037.
17. Nozaki, H., Misumi, O., Kuroiwa, T. Phylogeny of the quadriflagellate Volvocales (Chlorophyceae) based on chloroplast multigene sequences. *Molecular Phylogenetics and Evolution* **2003**, *29*, 58-66.
18. Morita, E., Abe, T., Tsuzuki, M., Fujiwara, S., Sato, N., Hirata, A., Nozaki, H. Role of pyrenoids in the CO 2-concentrating mechanism: comparative morphology, physiology and molecular phylogenetic analysis of closely related strains of *Chlamydomonas and Chloromonas* (Volvocales). *Planta* **1999**, *208*, 365-372.
19. Nozaki, H., Ito, M., Sano, R., Uchida, H., Watanabe, M. M., Takahashi, H., Kuroiwa, T. Phylogenetic analysis of *Yamagishlella* and *Platydorina* (Volvovaceae, Chlorophyta) based on rbcL gene sequences 1. *Journal of Phycology* **1997**, *33*, 272-278.
20. Sherwood, A.R., Garbary,D.J. Sheath,R.G. Assessing the phylogenetic position of the Prasiolales (Chlorophyta) using. *Phycologia* **2000**, *39*, 139-146.
21. Rindi, F., McIvor, L., Guiry, M. D. . The Prasiolales (chlorophyta) of Atlantic Europe: AN Assessment Based on Morphological, Molecular, and Ecological Data, Including the Characterization of Rosenvingiella Radicans (KÜTZING) Comb. NOV. 1. *Journal of Phycology* **2004**, *40*, 977-997.
22. Nakazawa, A., Krienitz, L., Nozaki, H. Taxonomy of the unicellular green algal genus *Vitreochlamys* (Volvocales), based on comparative morphology of cultured material. *European Journal of Phycology* **2001**, *36*, 113-128.

23. Zou, S., Fei, C., Song, J., Bao, Y., He, M., Wang, C. Combining and comparing coalescent, distance and character-based approaches for barcoding microalgaes: a test with *Chlorella*-like species (Chlorophyta). *PLoS One* **2016**, *11*, e0153833.
24. Kang, N.S., Lee, J. A., Jang, H. S., Kim, K. M., Kim, E. S., Yoon, M., Hong, J. W. First record of a marine microalgal species, *Chlorella gloriosa* (Trebouxiophyceae) isolated from the Dokdo Islands, Korea. *Korean Journal of Environmental Biology* **2019**, *37*, 526-534.
25. Kaplan-Levy, R.N., Alster-Gloukhovski, A., Benyamini, Y., Zohary, T. Lake Kinneret phytoplankton: integrating classical and molecular taxonomy. *Hydrobiologia* **2016**, *764*, 283-302.
26. Gomaa, F., Kosakyan, A., Heger, T. J., Corsaro, D., Mitchell, E. A., Lara, E. One alga to rule them all: unrelated mixotrophic testate amoebae (Amoebozoa, Rhizaria and Stramenopiles) share the same symbiont (Trebouxiophyceae). *Protist* **2014**, *165*, 161-176.
27. Amberg, S.M.M., R. H. Nucleotide sequence of the genes for ribulose-1,5-bisphosphate carboxylase/oxygenase large subunit and ribosomal protein S14 from a *Chlorella*-like green alga. *Journal of phycology* **1991**, *27*, 753-758.
28. Rindi, F., Mikhailyuk, T. I., Sluiman, H. J., Friedl, T., López-Bautista, J. M. Phylogenetic relationships in *Interfilum* and *Klebsormidium* (Klebsormidiophyceae, Streptophyta). *Molecular phylogenetics and evolution* **2011**, *58*, 218-231.
29. Neustupa, J., Němcová, Y., Vesela, J., Steinova, J., Škaloud, P. *Leptochlorella corticola* gen. et sp. nov. and *Kalinella apyrenoidosa* sp. nov.: two novel Chlorella-like green microalgae (Trebouxiophyceae, Chlorophyta) from subaerial habitats. *International journal of systematic and evolutionary microbiology* **2013**, *63*, 377-387.
30. Fawley, M.W., Fawley, K. P., Owen, H. A. Diversity and ecology of small coccoid green algae from Lake Itasca, Minnesota, USA, including Meyerella planktonica, gen. et sp. nov. *Phycologia* **2005**, *44*, 35-48.
31. Lobakova, E.S., Selyakh, I. O., Semenova, L. R., Scherbakov, P. N., Fedorenko, T. A., Chekanov, K. A., Gorelova, O. A. Hints for understanding microalgal phosphate-resilience from *Micractinium simplicissimum* IPPAS C-2056 (Trebouxiophyceae) isolated from a phosphorus-polluted site. *Journal of Applied Phycology* **2022**, *34*, 2409-2422.
32. Yakimovich, K.M., Gauthier, N. P., Engstrom, C. B., Leya, T., Quarmby, L. M. A molecular analysis of microalgae from around the globe to revise *Raphidonema* (Trebouxiophyceae, Chlorophyta). *Journal of phycology* **2021**, *57*, 1419-1432.
